# Pregnancy outcome documentation in low-resource settings: a multi-country study

**DOI:** 10.1016/j.xagr.2026.100696

**Published:** 2026-09-16

**Authors:** Alice Hucko, Jana Prauseová, Edward P.K. Parker, Rachel Ford, Oluwatosin Nkereuwem, Ugochukwu Madubueze, Suraj Bhattarai, Dan Kajungu, Agnes Msoka, Mir Mobarak Hossain, Beate Kampmann

**Affiliations:** 1 Centre for Global Health, Charité—Universitätsmedizin Berlin, Berlin, Germany (Hucko, Prauseová, Kampmann); 2 Department of Infectious Disease Epidemiology and International Health, London School of Hygiene & Tropical Medicine, London, UK (Parker); 3 Department of Clinical Research, London School of Hygiene & Tropical Medicine, London, UK (Ford and Kampmann); 4 School of Public Health, Faculty of Medicine, The University of Queensland, Brisbane, Queensland, Australia (Ford); 5 Medical Research Council Unit, The Gambia at the London School of Hygiene and Tropical Medicine, Fajara, The Gambia (Nkereuwem and Kampmann); 6 Alex-Ekwueme Federal University Teaching Hospital Abakaliki (AEFUTHA), Ebonyi State, Nigeria (Madubueze); 7 Alex-Ekwueme Federal University, Ndufu-Alike Ikwo, Ebonyi State, Nigeria (Madubueze); 8 Global Health Research and Medical Interventions Institute, Kathmandu, Nepal (Bhattarai); 9 Department of_Infectious Disease Epidemiology and International Health, London School of Hygiene & Tropical Medicine, London, UK (Bhattarai); 10 Makerere University Centre for Health and Population Research, Kampala, Uganda (Kajungu); 11 Division of Epidemiology and Biostatistics, Department of Global Health, Stellenbosch University, Stellenbosch, Cape Town, South Africa (Kajungu); 12 Kilimanjaro Clinical Research Institute (KCRI), Moshi, Tanzania (Msoka); 13 Kilimanjaro Christian Medical Centre (KCMCU), Moshi, Tanzania (KCMCU); 14 Kilimanjaro College of Health and Allied Sciences, School of Nursing, Moshi, Tanzania (Msoka); 15 Planning and Research Unit, Directorate General of Health Services, Dhaka, Bangladesh (Hossain)

## Abstract

**BACKGROUND:**

As the maternal vaccine portfolio expands, reliable documentation of adverse events of special interest (AESI) is critical for safety surveillance in low- and middle-income countries (LMIC). However, such documentation is often not easily available, even prior to introduction of new vaccines, and background rates are therefore lacking.

**METHODS:**

Nested within a retrospective multisite analysis of routine maternal health data from six LMICs (Bangladesh, The Gambia, Nepal, Nigeria, Tanzania, and Uganda) from 2019 to 2022, we extracted aggregated monthly counts of vaccination-relevant pregnancy AESI- preterm birth, stillbirth, congenital malformations, neonatal death, and maternal death- and assessed their availability, completeness, and consistency using descriptive analyses. Outcome rates were compared with published country-specific background estimates.

**FINDINGS:**

Among 123,867 pregnancies across 27 clinics (totaling 1277 clinic-months) in six LMICs, substantial limitations in pregnancy outcome recording were identified. AESI recording was predominantly paper-based (clinic registers in 92.6% of clinics, maternal-held documents in 51.9%); with use of digital systems in only 14.8% of clinics. Congenital malformations and preterm subcategories were absent in seven of 27 clinics (25.9%). Documentation completeness ranged from 65.2% to 68.9% for preterm birth categories to 96.8% to 98.4% for mortality outcomes (stillbirths, neonatal and maternal deaths). Considerable inter-clinic variability was noted, and observed rates diverged substantially from national background estimates for several pregnancy outcomes.

**INTERPRETATION:**

Substantial gaps in documentation of routine clinical data across LMIC underscore the need for linked, standardized, and digital maternal and neonatal outcome documentation to support the safe introduction and surveillance of vaccines administered during pregnancy.

**AJOG Global Reports at a Glance**

**A. Why was this study conducted?**

Reliable safety surveillance for vaccines given during pregnancy depends on robust documentation of pregnancy adverse events, but the quality of existing routine data in low- and middle-income countries (LMIC) is largely unknown.

**B. Key findings?**

Across 27 clinics in six LMIC and more than 120,000 pregnancies, outcome documentation was predominantly paper-based and inconsistent. Congenital malformations and preterm birth subcategories were entirely absent from records in one quarter of clinics. Observed outcome rates diverged substantially from published national background estimates.

**C. What does this add to what is known?**

This is the first multi-country, multisite assessment of routine pregnancy adverse event documentation framed around maternal vaccine safety surveillance needs. Findings show routine health data systems require improvements to support baseline safety monitoring before and after maternal vaccine introduction.

## Introduction

Pregnancy and early neonatal life are periods of heightened susceptibility to infectious diseases, most consequential in low- and middle-income countries (LMIC) where they remain leading causes of maternal^1^ and neonatal^2^ morbidity and mortality. Maternal vaccination is therefore a critical strategy to protect both mothers and newborns, with vaccines against tetanus, diphtheria, pertussis, influenza, COVID-19, respiratory syncytial virus (RSV), and Ebolavirus demonstrating effectiveness and an acceptable safety profile during pregnancy.^3^, ^4^, ^5^

The recent approval of a maternal RSV vaccine^6^ and ongoing development of a group B streptococcus (GBS) vaccine^7^ mark an important expansion of the maternal immunization portfolio, and their availability in LMIC is eagerly anticipated. Introducing these novel vaccines heightens the need for robust, context-specific safety data to support signal detection and background rate interpretation. This requires systematic recording of adverse events of special interest (AESI), in particular preterm birth, stillbirth, neonatal death, and maternal death.

Strengthening maternal vaccine safety assessments requires a clear understanding of existing surveillance capacity, data completeness, and the feasibility of estimating background rates within routine health systems. Context-specific evidence is particularly critical in LMICs, where the burden of RSV and other vaccine-preventable maternal and neonatal infections is highest, yet safety monitoring infrastructure may be comparatively limited.

In this study, we assess the availability, completeness, and consistency of routinely recorded pregnancy and infant outcomes across six LMIC settings, characterizing current surveillance capacity and identifying gaps to inform preparedness for safe maternal vaccine introduction.

## Materials and methods

### Study setting

These data were generated as a sub-study of a multi-country, multi-site retrospective longitudinal data extraction study. The full study design and protocol have been previously published.^8^

In summary, between January 2019 and December 2022, data were collected from selected sites in Bangladesh, The Gambia, Nepal, Nigeria, Tanzania, and Uganda. In each country, local coinvestigators identified four to five eligible clinics, including two urban and two rural facilities, with the option of an additional private clinic where feasible, to capture variation in healthcare settings and service delivery patterns. Fully anonymized secondary data were extracted and analyzed from routine maternal health records held at participating antenatal care (ANC) and delivery clinics over the 4-year period.

Ethical approval for this study was obtained from the Observational/Interventional Research Ethics Committee of the London School of Hygiene & Tropical Medicine and from the respective national ethics committees of all participating countries, in accordance with local regulatory requirements.

### Selection of indicators

This sub-study focused on pregnancy outcomes classified as AESI in the context of vaccination during pregnancy. These included late preterm birth, very preterm birth, extremely preterm birth, stillbirth, congenital malformations, neonatal death, and maternal death. Standardized case definitions developed by the Global Alignment on Immunization Safety Assessment in Pregnancy (GAIA) project were used.^9^

### Data extraction

Data extraction focused on background clinic infrastructure and aggregated service delivery data. Facility-level information was collected once at the start of the data collection process at each participating site to characterize the service delivery context. This included staffing composition, clinic opening times, and current practices of recording pregnancy outcomes and adverse events. Deidentified data were obtained through direct observation of the clinics and review of maternal health registers.

Monthly aggregated counts were derived from routine maternal health records maintained at participating clinics, including antenatal, delivery, and postnatal registers, as well as supplementary notebooks where available. These included the number of ANC attendances, number of deliveries, and counts of AESI by type.

All data were entered into country-specific REDCap databases using electronic data collection devices, such as tablets or smartphones. Data were subsequently extracted from the REDCap databases for analysis.

### Data analysis

We performed an initial descriptive analysis of facility-level characteristics overall and stratified by country. Using clinic-level data aggregated by month, we determined the completeness of pregnancy outcome recording over time. We first defined the number of months per year in which data were available for a given clinic based on a valid numeric recording for number of ANC visits, number of deliveries, or any pregnancy-related AESI. We then determined the proportion of months in which records were available for each pregnancy-related AESI. Finally, we quantified outcome rates for each pregnancy-related AESI stratified by country, clinic, and year. Numerator and denominator counts were derived from aggregated monthly data, excluding months in which data for a given AESI were not available. Binomial 95% confidence intervals were calculated using the Wilson Score method. As descriptive comparators, we used country-specific background rates from WHO, UNICEF and World Bank—available for neonatal mortality,^10^ maternal mortality,^11^ preterm birth,^12^ and stillbirth.^13^

## Results

### Study population and data coverage

Twenty-seven antenatal and delivery clinics across six countries contributed data to the study. After data filtering, 123,867 pregnancies were included in the analytic dataset: 33,950 from Bangladesh, 20,918 from The Gambia, 9645 from Nepal, 41,065 from Nigeria, 6332 from Tanzania, and 11,957 from Uganda.^8^ Clinics included 16 urban facilities (59.3%), eight rural facilities (29.6%), and three private facilities (11.1%) (Supplemental Table S1), with a total of 1277 clinic-months under observation (Supplemental Table S2).

### Documentation across record types

#### Pregnancy outcome documentation

All clinics documented pregnancy outcomes in clinic-held ANC and/or labor ward registers, with the source of documentation varying across countries and clinics (Figure 1). Pregnancy outcomes were documented in maternal-held records in 20/27 clinics (74.1%); overlapping documentation in both maternal and clinic-held records was common.

**Figure 1:**
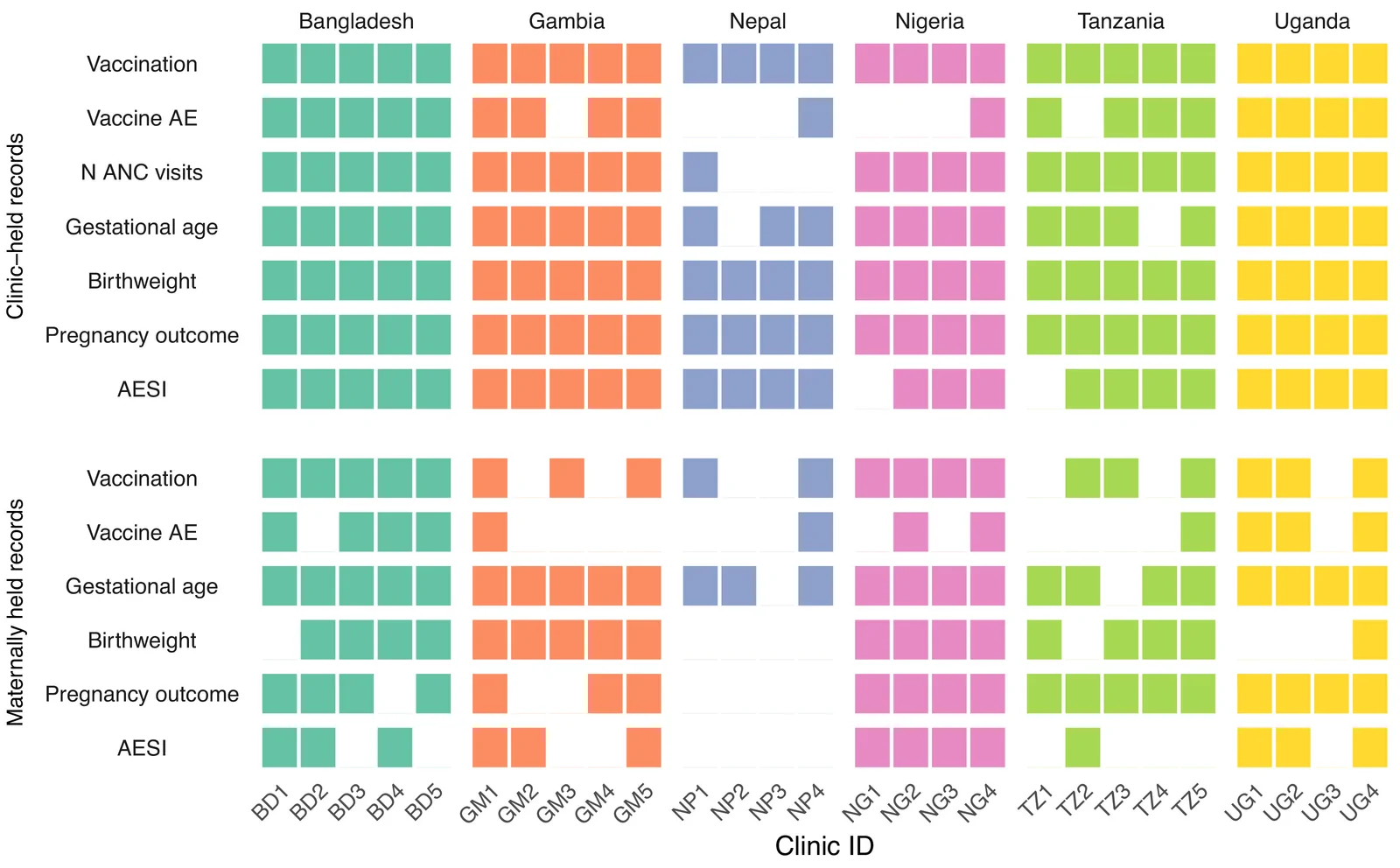
**Recording practices across clinics.** Data are presented as a heat map, wherein shaded squares indicate presence of a given characteristic. *AE*, adverse event; *AESI*, adverse event of special interest; *ANC*, antenatal care.

Hucko et al. Pregnancy outcome documentation in low-resource settings. AJOG Glob Rep 2026.

#### AESI documentation

The selected AESI indicators were documented in clinic-held paper records consisting of ANC paper registers and labor ward paper-based systems, as well as in maternal-held documents, and—in some cases- on digital platforms, with many clinics reporting documentation in more than one system. Twenty-five clinics (92.6%) recorded AESI in clinic-held registers, and 14 clinics (51.9%) recorded AESI in maternal-held documents. One clinic documented AESI exclusively in maternal-held documents. Only four clinics (14.8%) documented AESI in digital systems. Only one clinic lacked any form of AESI documentation.

#### Completeness of AESI documentation

The completeness of AESI documentation varied substantially between countries and outcome types (Figure 2). The largest gaps occurred in the documentation of congenital malformations and preterm birth subcategories. In The Gambia, no clinics recorded congenital malformations and no late, very, or extremely preterm births across the observation period. In Tanzania, three of five clinics lacked late preterm birth documentation, and two clinics had no records of congenital malformations or of very or extremely preterm births.

**Figure 2:**
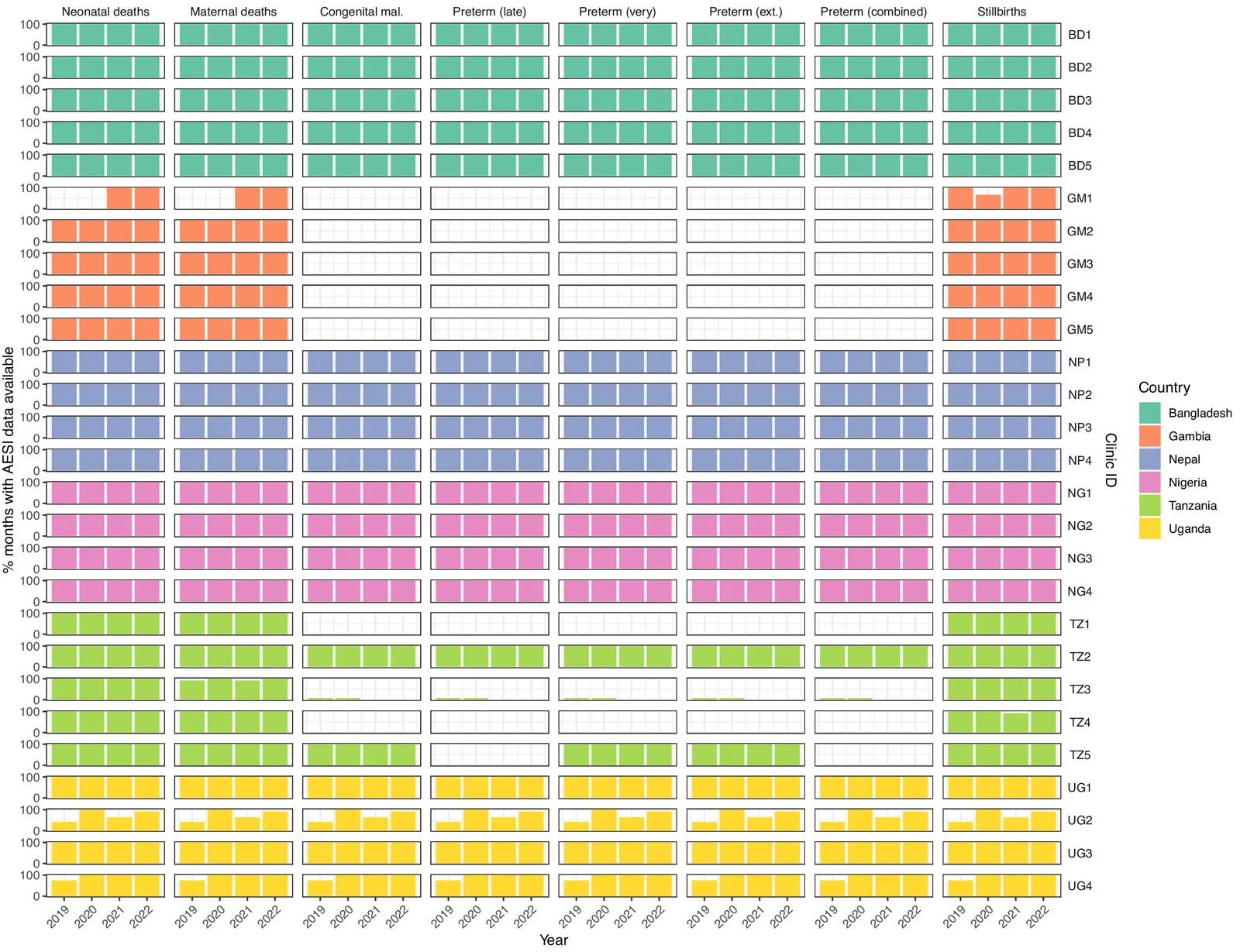
**Completeness of AESI documentation.** Data are presented by clinic and year. The number of months in a given year in which the clinic was open was used as a denominator, as inferred by the availability of data for ANC visit counts, delivery counts, or pregnancy outcome counts (see Supplemental Table S2).

Hucko et al. Pregnancy outcome documentation in low-resource settings. AJOG Glob Rep 2026.

Across the full set of 27 clinics, documentation of congenital malformations was absent in seven clinics, documentation of late preterm birth was absent in eight clinics, and documentation of very or extremely preterm births was absent in seven clinics. Outcome documentation was generally stable over time, with the exception of a single Gambian clinic in which neonatal and maternal death data were missing during 2019 to 2020.

Across all clinics combined, outcome documentation, defined as the proportion of clinic-months with available outcome data, was available for 98.4% of stillbirths, 96.9% of neonatal deaths, 96.8% of maternal deaths, 68.9% of congenital malformations, 68.9% of very preterm births, 68.9% of extremely preterm births, and 65.2% of late preterm births (Supplemental Table S2).

#### Observed vs published country-level reference AESI rates

Observed outcome rates differed markedly from publicly available country-level reference estimates^10^, ^11^, ^12^, ^13^ (Figure 3). Aggregating across clinics, neonatal mortality was lower than in country-level reference values across all six countries. Observed rates of late preterm birth were lower than reference estimates in four of five countries (with data lacking for this outcome among clinics in The Gambia). Observed rates for stillbirths were lower than reference estimates in Bangladesh, Tanzania, and Uganda, whereas observed rates were higher than reference values in Nigeria. Maternal mortality was also lower than reference estimates in all countries, with the smallest difference observed in Nepal and the largest in Nigeria, where the reference maternal mortality rate is approximately 1% of deliveries. Available country-level reference estimates report all preterm births as a single category (<37 weeks’ gestation), without further stratification by gestational age (GA).

**Figure 3:**
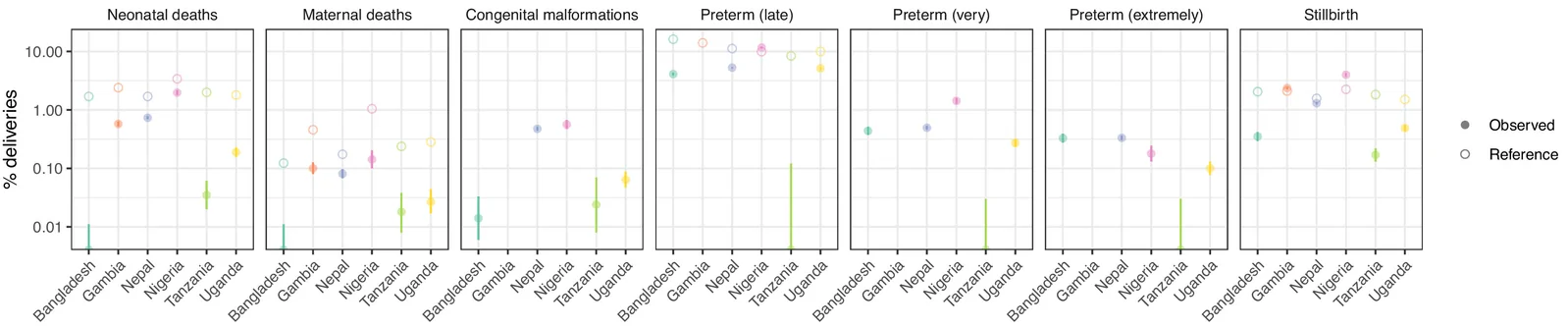
**AESI rates by country.** Observed rates are presented as closed circles. National reference rates are presented as open circles where available. WHO reference ranges refer to all prematurity under 37 weeks, without further stratification.

Hucko et al. Pregnancy outcome documentation in low-resource settings. AJOG Glob Rep 2026.

#### Temporal and inter-clinic variability

Outcome rates were broadly stable over time, with no consistent temporal trends during the 4-year observation period (Supplemental Figure S1). However, variation in outcome rates was observed between clinics within the same country. Variation was greatest in Nigeria, where all outcomes showed wide between-facility heterogeneity (Supplemental Figure S2). Within-country variability was also evident for neonatal and maternal deaths in The Gambia and Nepal, late preterm birth in Uganda, Nepal, and Bangladesh, and stillbirths in Bangladesh and The Gambia.

#### Prematurity assessments and GA documentation

All clinics assessed GA as the foundation for prematurity assessment using fundal height (Supplemental Table S1). Ultrasound was additionally used in 22 clinics (81.5%), and last menstrual period (LMP) was recorded in 23 clinics (85.2%). Approaches to fundal height measurement varied: four clinics (14.8%) measured fundal height by hand alone, 11 clinics (40.7%) used both hand and tape, and 12 clinics (44.4%) used tape only.

## Comment

This study analyzed routinely collected data from 123,867 pregnancies across 27 clinics in six low-resource settings and recorded pregnancy outcomes and AESI for maternal vaccine safety surveillance. Our results show substantial limitations in the availability, completeness, and consistency of routine ANC and delivery data: documentation of key outcomes, particularly congenital malformations and preterm birth subcategories, was frequently incomplete. AESI recording was fragmented, relying heavily on clinic-held paper registers and maternal-held documents, with limited digital capture. Marked between-clinic variability and sizable discrepancies between observed and reference rates were evident for several outcomes, including stillbirth, neonatal death, and maternal death.

Our findings highlight the fragmented nature of data recording across maternal-held records and clinic-held paper registers. Key outcomes such as AESI were inconsistently captured across these sources, oftentimes with no single record providing a comprehensive longitudinal account of pregnancy and delivery outcomes. Reliance on maternal-held documents introduces risks of data loss, incomplete transfer of information between facilities, and inequities for women with limited health literacy or unstable access to care. For maternal immunization programs, which require linkage between vaccination exposure, pregnancy course, and neonatal outcomes, the absence of integrated records represents a major structural barrier.^14^

Prematurity is a central AESI for maternal immunization safety surveillance and a key outcome for evaluating both anticipated benefits of vaccination and also potential harms during pregnancy. Under the GAIA framework, preterm birth and its subcategories (late, very, and extremely preterm) are defined AESIs that require accurate and reliable GA determination to allow meaningful safety assessment and comparability across settings.^15^ Robust GA assessment is therefore foundational to maternal vaccine surveillance. In this study, we identified substantial gaps in the availability, completeness, and consistency of prematurity data. These gaps arise from both limitations in GA assessment methods and deficiencies in the documentation of preterm birth outcomes.

All clinics assessed GA using fundal height, but measurement approaches varied considerably, including hand-based estimation, tape measurement, or both, potentially reducing precision where it serves as the primary dating method. Although ultrasound was reportedly available in most clinics, its routine use could not be confirmed and is unlikely to be universally accessible, particularly in LMIC settings where access is often restricted by cost, risk status, staffing, equipment, or late ANC presentation. LMP was also commonly recorded. Reliance on LMP and fundal height for GA dating may give rise to inaccuracies in prematurity assessment; multiple publications have demonstrated issues around reliability of LMP for GA dating, especially in contexts with irregular cycles and delayed care-seeking.^16^, ^17^, ^18^

Accuracy of GA dating is particularly important in the context of maternal immunization. GA influences both the timing of vaccination and efficiency of transplacental antibody transfer and, consequently, the degree of neonatal protection conferred. Jasset et al demonstrated that administration of the maternal RSV vaccine earlier in the approved 32- to 36-week window resulted in the highest transplacental transfer of maternal antibodies to the neonate. This underscores that optimal vaccine timing for maximal neonatal protection depends critically on reliable GA determination.^19^

The inconsistent application of multiple GA assessment methods with known limitations also compromises accurate and standardized classification of preterm birth.^15^ In our study, these challenges were compounded by gaps in the documentation of prematurity itself. Incomplete recording, particularly the absence of reliable differentiation between late, very, and extremely preterm birth, emerged as a consistent limitation across sites. A further contributing factor may be variability in GA thresholds for viability across sites, below which deliveries are classified and documented as abortions or pregnancy losses rather than live preterm births. This could not be assessed in the present study but may have contributed to systematic undercounting of very and extremely preterm births. Marked between-clinic variability in reported prematurity rates within the same countries further suggests inconsistencies in definitions, recording practices, or both. However, this variability may also reflect differences in the catchment populations served by individual clinics.

These documentation gaps carry direct implications for the evaluation of maternal vaccines. Prematurity acts simultaneously as a key safety endpoint and a potential efficacy outcome, both of which require granular, standardized GA-based classification to be meaningfully assessed. Regarding efficacy, Giles et al^20^ found that women who received both influenza and pertussis vaccines had 61% lower odds of preterm birth before 32 weeks than unvaccinated women. Similarly, GBS infection has been implicated in colonization and infection-associated preterm birth, raising the possibility that effective maternal GBS vaccination could reduce prematurity rates by preventing colonization or ascending or intrauterine infection.^21^ Without accurate and complete prematurity documentation, such potential benefits are unlikely to be detected, quantified, or attributed, limiting the ability to fully evaluate the vaccine impact.

In this context, the limitations extend beyond individual facilities to the national level: reference estimates typically aggregate all preterm births into a single category (<37 weeks’ gestation), likely reflecting the absence of systematically collected granular data. Comparative analyses were not feasible for very preterm birth and extremely preterm birth because national reference estimates for these outcomes were unavailable. The lack of stratified national baseline rates therefore precludes meaningful comparison across sites and substantially constrains robust safety surveillance and interpretation.

Documentation of congenital malformations was also frequently incomplete or absent across sites. This is consistent with wider literature from low-resource settings, where congenital anomaly recording and classification is often limited, and baseline rates are lacking.^22^^,^^23^ Although congenital anomalies are generally less likely to be causally associated with vaccines recommended in the second or third trimester, they remain important outcomes for surveillance and communication. This is particularly relevant during early vaccine rollout phases, when public confidence is most fragile, as transparent communication of safety information is essential to prevent misperceptions that may affect vaccine acceptance and uptake. Comparative assessment was also not possible due to the absence of national reference estimates for these congenital malformations.

Marked discrepancies between observed outcome frequencies and available country-level reference estimates were noted for several AESI, including prematurity, neonatal mortality, stillbirth, and maternal death. These findings contrast with previous multi-country research that estimated site-specific rates for preterm birth, low birthweight, neonatal death, and stillbirth across 21 geographically diverse sites and compared them with national-level estimates from WHO and UNICEF.^24^ In that study, site-specific outcome rates generally fell within the range of available national estimates, suggesting closer agreement between local and national data sources than observed in the present analysis. However, our findings are consistent with evidence from 31 Health and Demographic Surveillance System (HDSS) sites across sub-Saharan Africa and Asia, where substantial variability in pregnancy outcome reporting and likely under-ascertainment of adverse outcomes were identified, with lower neonatal mortality rates than national averages in most HDSS sites and under-recording of stillbirths compared to neonatal deaths in several sites.^25^ Similar challenges have also been described for maternal mortality estimation in Papua New Guinea, where substantial discrepancies were observed between national routine data, demographic survey findings, and internationally modelled estimates.^26^

The higher observed rates for certain pregnancy outcomes in some settings illustrate the potential for misinterpretation in the absence of robust, locally appropriate reference values. Conversely, the generally lower facility-based rates observed for neonatal mortality, maternal mortality, and late preterm birth raise concern about under-ascertainment or incomplete documentation of adverse outcomes in routine clinical data, which could obscure true background risk and reduce the sensitivity of postintroduction safety surveillance. Of note, as our data were derived from institutional sources, the reported maternal mortality rates may be lower than national estimates, which typically include out-of-facility births and are therefore more likely to capture higher-risk cases not reflected in facility-based data. Lastly, the discrepant rates could also be due to selection bias in participating sites.

The observed discrepancies in this analysis underscore the challenges of relying on paper-based records and reinforce concerns regarding the completeness and reliability of routine maternal and neonatal outcome data in LMIC settings. These findings highlight the need to strengthen local data systems to support harmonized, consistent, and accurate documentation for reliable background rate estimation in maternal vaccine safety surveillance.

Effective active surveillance further requires individual-level, longitudinal follow-up of both the mother and infant across pregnancy, delivery, and the postnatal period. However, such continuity is difficult to achieve within routine paper-based systems, which are often fragmented and poorly linked.^27^^,^^28^ Electronic medical records may offer a pathway to addressing this structural limitation by supporting longitudinal capture of maternal and neonatal data, while facilitating linkage between vaccination exposure, pregnancy course, and birth outcomes across points of care.^28^

This study has several strengths. It addresses a critical evidence gap by providing real-world, facility-based data on the documentation of over 120,000 pregnancy outcomes relevant to maternal vaccine safety surveillance across multiple clinics in six low-income countries. The data reflect routine recording practices within existing health systems rather than within the controlled conditions of clinical trials, offering an important insight into existing surveillance capacity. In addition, the 4-year observation period allows assessment of the consistency and stability of outcome documentation over time, including during periods of disruption such as the COVID-19 pandemic.

Certain limitations should be acknowledged. The data might not be nationally representative, as they originate from a limited number of clinics in selected regions of each country, and variation in clinic type and capacity may influence documentation practices. Temporary clinic closures and damaged, missing, or unavailable registers resulted in periods without outcome data. As data were aggregated at the clinic level, individual-level follow-up was not possible, limiting the ability to link outcomes across pregnancy or to distinguish true absence of events from absence of documentation. The selection of clinics for this study by investigators might have introduced bias toward better resourced settings, although urban and rural facilities were included.

## Conclusion

Collectively, our data point to significant limitations in the readiness of routine clinical data systems to support maternal vaccine safety monitoring in many LMIC settings. Our findings highlight the need for targeted investments in standardized documentation and surveillance approaches that are feasible within routine ANC workflows. Strengthening routine data systems, improving linkage between ANC, delivery records and infant records, prioritizing core AESI, and establishing locally relevant baseline rates are critical for meaningful interpretation of AESI and the safe, evidence-based, and trusted use of vaccines in pregnancy.

## Declaration of generative AI and AI-assisted technologies in the writing process

During the preparation of this work, the authors used ChatGPT and ClaudeAI for polishing tone and clarity of language. The authors reviewed and edited the output as needed and take full responsibility for the content of the published article.

## CRediT authorship contribution statement

**Alice Hucko:** Writing – original draft, Formal analysis, Data curation, Conceptualization. **Jana Prauseová:** Writing – original draft, Project administration, Formal analysis, Conceptualization. **Edward P.K. Parker:** Writing – review & editing, Visualization, Methodology, Formal analysis. **Rachel Ford:** Writing – review & editing, Project administration, Methodology, Investigation, Data curation. **Oluwatosin Nkereuwem:** Writing – review & editing, Project administration, Investigation, Data curation. **Ugochukwu Madubueze:** Writing – review & editing, Project administration, Investigation, Conceptualization. **Suraj Bhattarai:** Writing – review & editing, Validation, Investigation, Conceptualization. **Dan Kajungu:** Writing – review & editing, Project administration, Investigation, Data curation. **Agnes Msoka:** Writing – review & editing, Project administration, Investigation, Conceptualization. **Mir Mobarak Hossain:** Writing – review & editing, Resources, Project administration, Investigation, Data curation. **Beate Kampmann:** Writing – original draft, Supervision, Resources, Project administration, Methodology, Investigation, Funding acquisition, Formal analysis, Data curation, Conceptualization.

## Appendix Supplementary materials

Image, application 1
