# Supplementary material for: Pregnancy outcome documentation in low-resource settings: a multi-country study
Source: AJOG Glob Rep. 2026 Sep 16;6(4):100696. doi: 10.1016/j.xagr.2026.100696 (PMC13677763; doi:10.1016/j.xagr.2026.100696)
Supplement: Supplementary file 1 [file mmc1.pdf]

Supplementary material

Contents

P2 Supplementary Figure S1. Pregnancy outcome rates by year.
P3 Supplementary Figure S2. Pregnancy outcome rates by clinic.
P4 Supplementary Table S1. Clinic characteristics
P6 Supplementary Table S2. Months of outcome reporting by clinic and year
P9 Supplementary Table S3. AESI by clinic and year

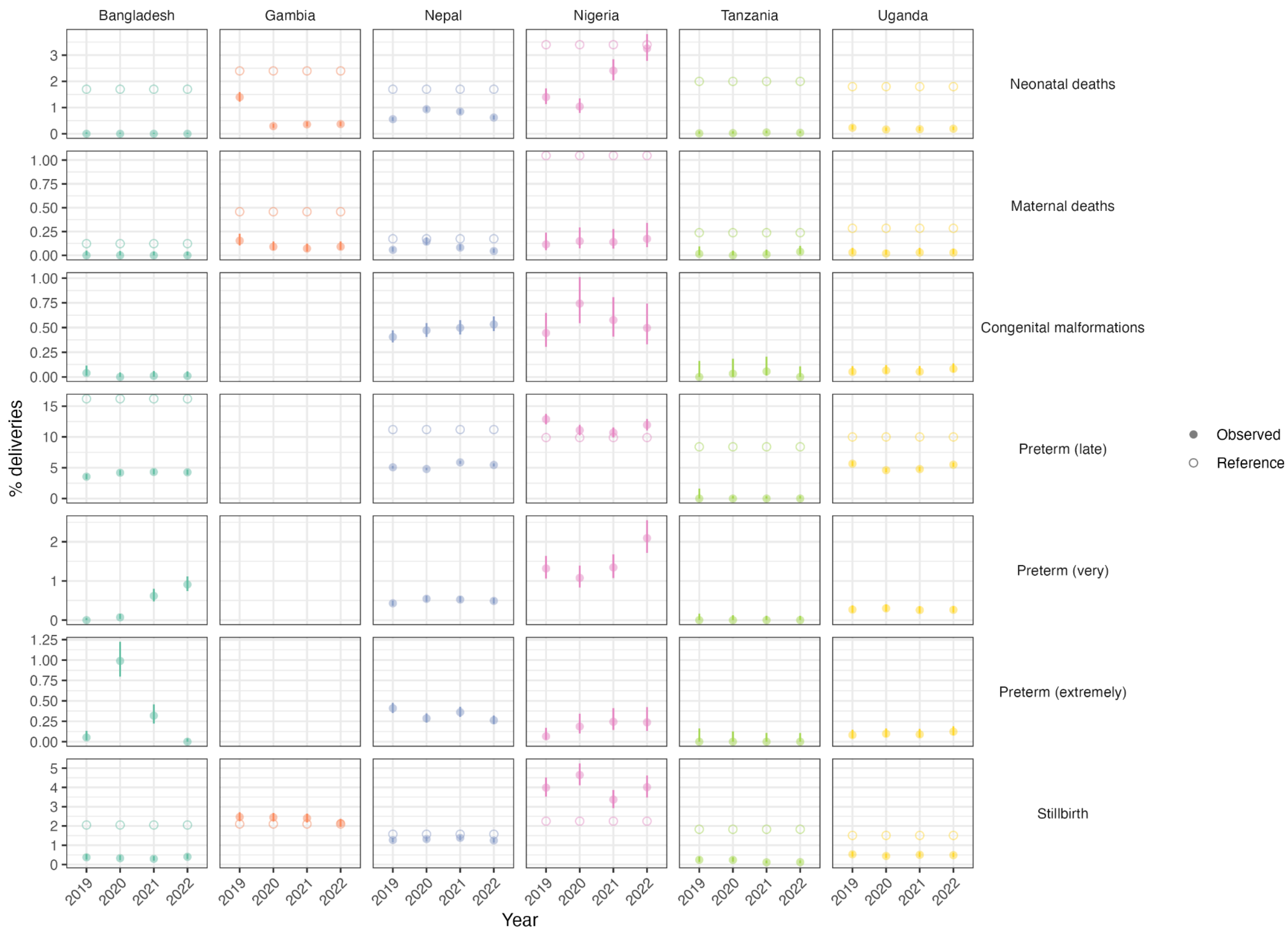

Supplementary Figure S1. **Pregnancy outcome rates by year.** National reference rates are presented as open circles where available.

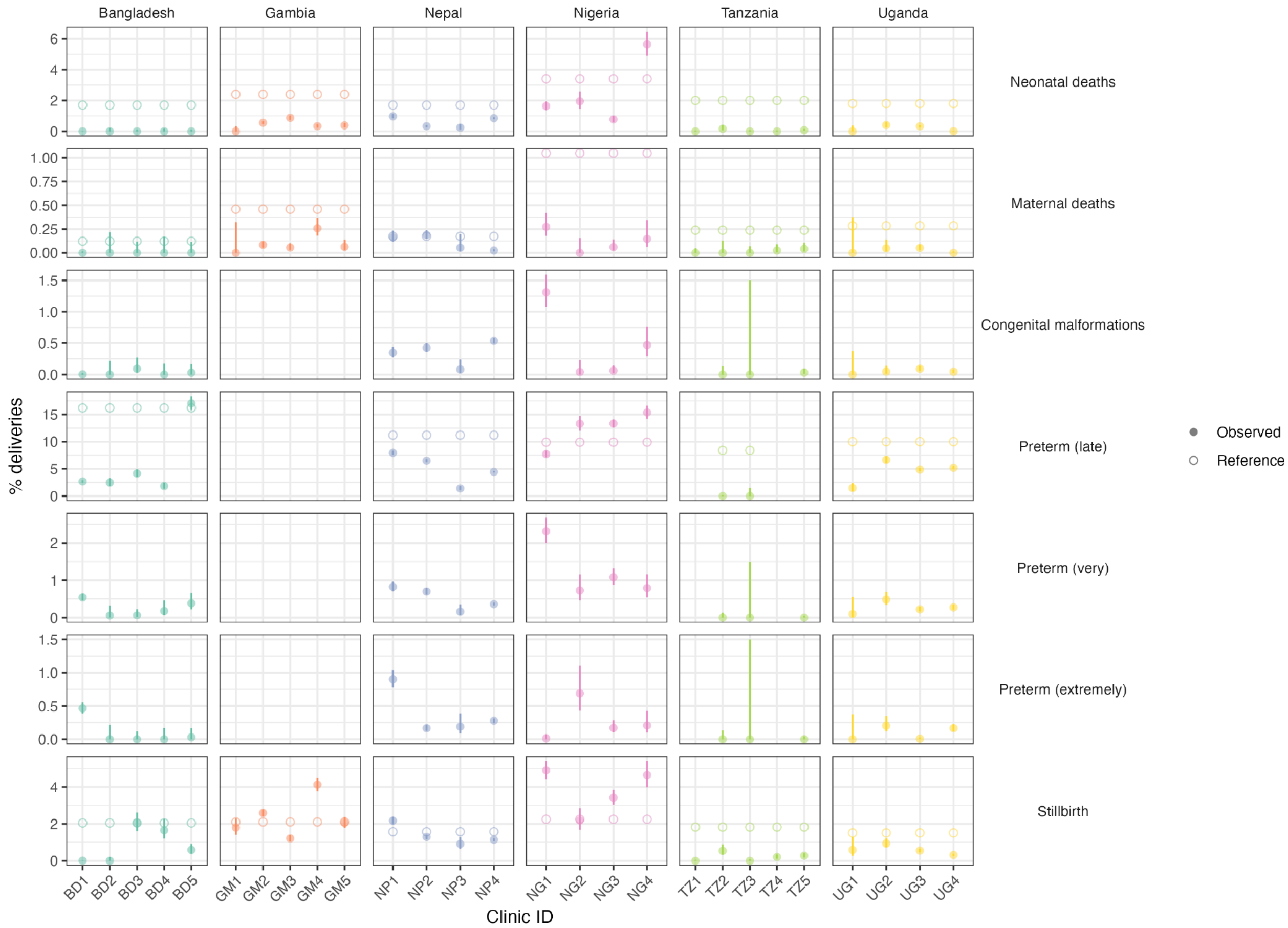

Supplementary Figure S2. Pregnancy outcome rates by clinic. National reference rates are presented as open circles where available.

Supplementary Table S1. Clinic characteristics

		Overall	Bangladesh	Gambia	Nepal	Nigeria	Tanzania	Uganda
**Variable**	**Value**	(N=27)	(N=5)	(N=5)	(N=4)	(N=4)	(N=5)	(N=4)
**N monthly ANC visits**	**Median (IQR)**	483 (191-1100)	214 (154-705)	616 (336-831)	470 (147-3691)	976 (564-1312)	330 (178-442)	1020 (171-2093)
**N monthly deliveries**	**Median (IQR)**	157 (74-428)	64 (43-91)	268 (142-448)	542 (154-1192)	116 (70-167)	140 (97-195)	480 (28-581)
**Setting**	**Private**	3 (11.1%)	1 (20.0%)	1 (20.0%)	0 (0%)	1 (25.0%)	0 (0%)	0 (0%)
	**Rural**	8 (29.6%)	2 (40.0%)	2 (40.0%)	0 (0%)	1 (25.0%)	2 (40.0%)	1 (25.0%)
	**Urban**	16 (59.3%)	2 (40.0%)	2 (40.0%)	4 (100%)	2 (50.0%)	3 (60.0%)	3 (75.0%)
**N clinics per week**	**Median (min-max)**	5 (2-7)	6 (6-7)	5 (4-6)	4 (2-6)	5 (2-5)	5 (5-5)	5 (4-6)
**Piped water**	**Yes**	26 (96.3%)	5 (100%)	4 (80.0%)	4 (100%)	4 (100%)	5 (100%)	4 (100%)
**Electricity**	**Mains and generator**	21 (77.8%)	4 (80.0%)	5 (100%)	3 (75.0%)	3 (75.0%)	2 (40.0%)	4 (100%)
	**Mains only**	5 (18.5%)	1 (20.0%)	0 (0%)	1 (25.0%)	0 (0%)	3 (60.0%)	0 (0%)
	**Generator only**	1 (3.7%)	0 (0%)	0 (0%)	0 (0%)	1 (25.0%)	0 (0%)	0 (0%)
**ANC clinic opening times**	**AM**	20 (74.1%)	3 (60.0%)	5 (100%)	0 (0%)	3 (75.0%)	5 (100%)	4 (100%)
	**AM/PM**	7 (25.9%)	2 (40.0%)	0 (0%)	4 (100%)	1 (25.0%)	0 (0%)	0 (0%)
**N ANC staff per day**	**1 to 3**	11 (40.7%)	2 (40.0%)	2 (40.0%)	2 (50.0%)	0 (0%)	4 (80.0%)	1 (25.0%)
	**4 to 6**	4 (14.8%)	0 (0%)	2 (40.0%)	0 (0%)	0 (0%)	1 (20.0%)	1 (25.0%)
	**7 to 9**	5 (18.5%)	2 (40.0%)	1 (20.0%)	0 (0%)	0 (0%)	0 (0%)	2 (50.0%)
	**10+**	7 (25.9%)	1 (20.0%)	0 (0%)	2 (50.0%)	4 (100%)	0 (0%)	0 (0%)
**ANC staff**	**Midwife**	23 (85.2%)	4 (80.0%)	5 (100%)	1 (25.0%)	4 (100%)	5 (100%)	4 (100%)
	**Nurse**	19 (70.4%)	3 (60.0%)	3 (60.0%)	3 (75.0%)	4 (100%)	5 (100%)	1 (25.0%)
	**Doctor**	18 (66.7%)	5 (100%)	2 (40.0%)	2 (50.0%)	4 (100%)	3 (60.0%)	2 (50.0%)
	**Community HW**	5 (18.5%)	1 (20.0%)	2 (40.0%)	0 (0%)	2 (50.0%)	0 (0%)	0 (0%)
	**Obstetrician**	16 (59.3%)	5 (100%)	2 (40.0%)	2 (50.0%)	4 (100%)	1 (20.0%)	2 (50.0%)
	**Other**	9 (33.3%)	3 (60.0%)	1 (20.0%)	0 (0%)	3 (75.0%)	2 (40.0%)	0 (0%)
**Standard of midwife care**	**Comprehensive EmONC**	23 (85.2%)	5 (100%)	3 (60.0%)	4 (100%)	4 (100%)	3 (60.0%)	4 (100%)
**Who discusses pregnancy vaccines**	**Midwife**	17 (63.0%)	3 (60.0%)	3 (60.0%)	1 (25.0%)	1 (25.0%)	5 (100%)	4 (100%)
	**Nurse**	14 (51.9%)	3 (60.0%)	1 (20.0%)	1 (25.0%)	4 (100%)	5 (100%)	0 (0%)
	**Doctor**	7 (25.9%)	3 (60.0%)	0 (0%)	2 (50.0%)	1 (25.0%)	1 (20.0%)	0 (0%)
	**Community health worker**	1 (3.7%)	0 (0%)	0 (0%)	0 (0%)	1 (25.0%)	0 (0%)	0 (0%)
	**Other**	5 (18.5%)	3 (60.0%)	2 (40.0%)	0 (0%)	0 (0%)	0 (0%)	0 (0%)
**Tetanus toxoid location**	**ANC/EPI**	9 (33.3%)	1 (20.0%)	0 (0%)	1 (25.0%)	4 (100%)	3 (60.0%)	0 (0%)
	**EPI**	8 (29.6%)	4 (80.0%)	4 (80.0%)	0 (0%)	0 (0%)	0 (0%)	0 (0%)
	**ANC**	10 (37.0%)	0 (0%)	1 (20.0%)	3 (75.0%)	0 (0%)	2 (40.0%)	4 (100%)
**Vaccination appointment with ANC**	**Either**	4 (14.8%)	3 (60.0%)	0 (0%)	0 (0%)	1 (25.0%)	0 (0%)	0 (0%)
	**Same routine ANC appointment**	22 (81.5%)	1 (20.0%)	5 (100%)	4 (100%)	3 (75.0%)	5 (100%)	4 (100%)
	**Separate appointment**	1 (3.7%)	1 (20.0%)	0 (0%)	0 (0%)	0 (0%)	0 (0%)	0 (0%)
**Who administers tetanus toxoid vaccine**	**Midwife**	11 (40.7%)	1 (20.0%)	0 (0%)	1 (25.0%)	0 (0%)	5 (100%)	4 (100%)
	**Nurse**	14 (51.9%)	2 (40.0%)	1 (20.0%)	2 (50.0%)	4 (100%)	5 (100%)	0 (0%)
	**EPI worker**	9 (33.3%)	4 (80.0%)	4 (80.0%)	1 (25.0%)	0 (0%)	0 (0%)	0 (0%)
	**Community health worker**	1 (3.7%)	0 (0%)	0 (0%)	0 (0%)	1 (25.0%)	0 (0%)	0 (0%)
	**Other**	2 (7.4%)	2 (40.0%)	0 (0%)	0 (0%)	0 (0%)	0 (0%)	0 (0%)
**Formal tool for recording vaccines in pregnancy**	**Yes**	25 (92.6%)	5 (100%)	4 (80.0%)	4 (100%)	4 (100%)	4 (80.0%)	4 (100%)

**Who records vaccines in pregnancy**	**Midwife**	11 (40.7%)	1 (20.0%)	1 (20.0%)	1 (25.0%)	0 (0%)	4 (80.0%)	4 (100%)
	**Nurse**	11 (40.7%)	1 (20.0%)	1 (20.0%)	2 (50.0%)	3 (75.0%)	4 (80.0%)	0 (0%)
	**EPI worker**	9 (33.3%)	4 (80.0%)	4 (80.0%)	1 (25.0%)	0 (0%)	0 (0%)	0 (0%)
	**Other**	3 (11.1%)	2 (40.0%)	0 (0%)	0 (0%)	1 (25.0%)	0 (0%)	0 (0%)
**Recording format for vaccines in pregnancy**	**Digital**	4 (14.8%)	1 (20.0%)	0 (0%)	0 (0%)	1 (25.0%)	1 (20.0%)	1 (25.0%)
	**Paper**	27 (100%)	5 (100%)	5 (100%)	4 (100%)	4 (100%)	5 (100%)	4 (100%)
	**Maternal personal**	20 (74.1%)	5 (100%)	3 (60.0%)	2 (50.0%)	4 (100%)	3 (60.0%)	3 (75.0%)
	**Other**	1 (3.7%)	0 (0%)	0 (0%)	1 (25.0%)	0 (0%)	0 (0%)	0 (0%)
**AE recorded for vaccines in pregnancy**	**Yes**	20 (74.1%)	5 (100%)	4 (80.0%)	1 (25.0%)	2 (50.0%)	4 (80.0%)	4 (100%)
**AE recording format for vaccines in pregnancy**	**Digital**	3 (11.1%)	1 (20.0%)	0 (0%)	0 (0%)	0 (0%)	1 (20.0%)	1 (25.0%)
	**Paper**	19 (70.4%)	5 (100%)	4 (80.0%)	1 (25.0%)	1 (25.0%)	4 (80.0%)	4 (100%)
	**Maternal personal**	12 (44.4%)	4 (80.0%)	1 (20.0%)	1 (25.0%)	2 (50.0%)	1 (20.0%)	3 (75.0%)
	**Other**	2 (7.4%)	0 (0%)	1 (20.0%)	0 (0%)	0 (0%)	1 (20.0%)	0 (0%)
**ANC dates recorded**	**Yes**	27 (100%)	5 (100%)	5 (100%)	4 (100%)	4 (100%)	5 (100%)	4 (100%)
**N ANC visits recorded**	**Yes**	27 (100%)	5 (100%)	5 (100%)	4 (100%)	4 (100%)	5 (100%)	4 (100%)
**N ANC visits recording format**	**Digital**	6 (22.2%)	2 (40.0%)	0 (0%)	0 (0%)	2 (50.0%)	2 (40.0%)	0 (0%)
	**Paper**	24 (88.9%)	5 (100%)	5 (100%)	1 (25.0%)	4 (100%)	5 (100%)	4 (100%)
	**ANC card**	27 (100%)	5 (100%)	5 (100%)	4 (100%)	4 (100%)	5 (100%)	4 (100%)
**Gestational age measurement method**	**Ultrasound**	22 (81.5%)	5 (100%)	4 (80.0%)	2 (50.0%)	4 (100%)	5 (100%)	2 (50.0%)
	**Fundal**	27 (100%)	5 (100%)	5 (100%)	4 (100%)	4 (100%)	5 (100%)	4 (100%)
	**LMP**	23 (85.2%)	5 (100%)	1 (20.0%)	4 (100%)	4 (100%)	5 (100%)	4 (100%)
**Fundal height measurement method**	**Hand**	4 (14.8%)	2 (40.0%)	0 (0%)	1 (25.0%)	0 (0%)	0 (0%)	1 (25.0%)
	**Hand/Tape**	11 (40.7%)	3 (60.0%)	1 (20.0%)	3 (75.0%)	1 (25.0%)	0 (0%)	3 (75.0%)
	**Tape**	12 (44.4%)	0 (0%)	4 (80.0%)	0 (0%)	3 (75.0%)	5 (100%)	0 (0%)
**Gestational age recording format**	**ANC register (digital)**	6 (22.2%)	2 (40.0%)	0 (0%)	0 (0%)	2 (50.0%)	2 (40.0%)	0 (0%)
	**ANC register (paper)**	25 (92.6%)	5 (100%)	5 (100%)	3 (75.0%)	4 (100%)	4 (80.0%)	4 (100%)
	**Maternal personal record**	25 (92.6%)	5 (100%)	5 (100%)	3 (75.0%)	4 (100%)	4 (80.0%)	4 (100%)
	**Labour ward register (paper)**	7 (25.9%)	1 (20.0%)	0 (0%)	1 (25.0%)	2 (50.0%)	1 (20.0%)	2 (50.0%)
**Pregnancy outcome recording format**	**ANC register (digital)**	3 (11.1%)	1 (20.0%)	0 (0%)	0 (0%)	1 (25.0%)	1 (20.0%)	0 (0%)
	**ANC register (paper)**	8 (29.6%)	2 (40.0%)	2 (40.0%)	0 (0%)	1 (25.0%)	1 (20.0%)	2 (50.0%)
	**Maternal personal record**	20 (74.1%)	4 (80.0%)	3 (60.0%)	0 (0%)	4 (100%)	5 (100%)	4 (100%)
	**Labour ward register (digital)**	3 (11.1%)	1 (20.0%)	0 (0%)	0 (0%)	1 (25.0%)	1 (20.0%)	0 (0%)
	**Labour ward register (paper)**	25 (92.6%)	4 (80.0%)	4 (80.0%)	4 (100%)	4 (100%)	5 (100%)	4 (100%)
**Birthweight recording format**	**Digital**	5 (18.5%)	2 (40.0%)	0 (0%)	0 (0%)	1 (25.0%)	1 (20.0%)	1 (25.0%)
	**Paper**	27 (100%)	5 (100%)	5 (100%)	4 (100%)	4 (100%)	5 (100%)	4 (100%)
	**Maternal ANC**	18 (66.7%)	4 (80.0%)	5 (100%)	0 (0%)	4 (100%)	4 (80.0%)	1 (25.0%)
	**Infant card**	27 (100%)	5 (100%)	5 (100%)	4 (100%)	4 (100%)	5 (100%)	4 (100%)
**AESI recording format**	**ANC register (digital)**	2 (7.4%)	1 (20.0%)	0 (0%)	0 (0%)	1 (25.0%)	0 (0%)	0 (0%)
	**ANC register (paper)**	12 (44.4%)	3 (60.0%)	4 (80.0%)	0 (0%)	1 (25.0%)	2 (40.0%)	2 (50.0%)
	**Maternal personal record**	14 (51.9%)	3 (60.0%)	3 (60.0%)	0 (0%)	4 (100%)	1 (20.0%)	3 (75.0%)
	**Labour ward register (digital)**	2 (7.4%)	0 (0%)	0 (0%)	0 (0%)	0 (0%)	1 (20.0%)	1 (25.0%)
	**Labour ward register (paper)**	22 (81.5%)	3 (60.0%)	4 (80.0%)	4 (100%)	3 (75.0%)	4 (80.0%)	4 (100%)

AE, adverse event; AESI, adverse event of special interest; ANC, antenatal clinic; EmONC, Emergency Obstetric and Newborn Care; EPI, Expanded Programme on Immunization; IQR, interquartile range; LMP, last menstrual period.

Supplementary Table S2. Months of outcome reporting by clinic and year

				N months reporting on outcome						
**Country**	**Clinic ID**	**Year**	**N months open***	**Neonatal deaths**	**Maternal deaths**	**Congenital malformations**	**Late preterm**	**Very preterm**	**Extremely preterm**	**Stillbirth**
Bangladesh	BD1	2019	12	12	12	12	12	12	12	12
Bangladesh	BD1	2020	12	12	12	12	12	12	12	12
Bangladesh	BD1	2021	12	12	12	12	12	12	12	12
Bangladesh	BD1	2022	12	12	12	12	12	12	12	12
Bangladesh	BD2	2019	12	12	12	12	12	12	12	12
Bangladesh	BD2	2020	12	12	12	12	12	12	12	12
Bangladesh	BD2	2021	12	12	12	12	12	12	12	12
Bangladesh	BD2	2022	12	12	12	12	12	12	12	12
Bangladesh	BD3	2019	12	12	12	12	12	12	12	12
Bangladesh	BD3	2020	12	12	12	12	12	12	12	12
Bangladesh	BD3	2021	12	12	12	12	12	12	12	12
Bangladesh	BD3	2022	12	12	12	12	12	12	12	12
Bangladesh	BD4	2019	12	12	12	12	12	12	12	12
Bangladesh	BD4	2020	12	12	12	12	12	12	12	12
Bangladesh	BD4	2021	12	12	12	12	12	12	12	12
Bangladesh	BD4	2022	12	12	12	12	12	12	12	12
Bangladesh	BD5	2019	12	12	12	12	12	12	12	12
Bangladesh	BD5	2020	12	12	12	12	12	12	12	12
Bangladesh	BD5	2021	12	12	12	12	12	12	12	12
Bangladesh	BD5	2022	12	12	12	12	12	12	12	12
Gambia	GM1	2019	12	0	0	0	0	0	0	12
Gambia	GM1	2020	12	0	0	0	0	0	0	8
Gambia	GM1	2021	12	12	12	0	0	0	0	12
Gambia	GM1	2022	12	12	12	0	0	0	0	12
Gambia	GM2	2019	12	12	12	0	0	0	0	12
Gambia	GM2	2020	12	12	12	0	0	0	0	12
Gambia	GM2	2021	12	12	12	0	0	0	0	12
Gambia	GM2	2022	12	12	12	0	0	0	0	12
Gambia	GM3	2019	12	12	12	0	0	0	0	12
Gambia	GM3	2020	12	12	12	0	0	0	0	12
Gambia	GM3	2021	12	12	12	0	0	0	0	12
Gambia	GM3	2022	12	12	12	0	0	0	0	12
Gambia	GM4	2019	12	12	12	0	0	0	0	12
Gambia	GM4	2020	12	12	12	0	0	0	0	12
Gambia	GM4	2021	12	12	12	0	0	0	0	12
Gambia	GM4	2022	12	12	12	0	0	0	0	12
Gambia	GM5	2019	12	12	12	0	0	0	0	12
Gambia	GM5	2020	12	12	12	0	0	0	0	12
Gambia	GM5	2021	12	12	12	0	0	0	0	12
Gambia	GM5	2022	12	12	12	0	0	0	0	12
Nepal	NP1	2019	12	12	12	12	12	12	12	12
Nepal	NP1	2020	12	12	12	12	12	12	12	12
Nepal	NP1	2021	12	12	12	12	12	12	12	12

				N months reporting on outcome						
**Country**	**Clinic ID**	**Year**	**N months open***	**Neonatal deaths**	**Maternal deaths**	**Congenital malformations**	**Late preterm**	**Very preterm**	**Extremely preterm**	**Stillbirth**
Bangladesh	BD1	2019	12	12	12	12	12	12	12	12
Bangladesh	BD1	2020	12	12	12	12	12	12	12	12
Nepal	NP1	2022	12	12	12	12	12	12	12	12
Nepal	NP2	2019	12	12	12	12	12	12	12	12
Nepal	NP2	2020	12	12	12	12	12	12	12	12
Nepal	NP2	2021	12	12	12	12	12	12	12	12
Nepal	NP2	2022	12	12	12	12	12	12	12	12
Nepal	NP3	2019	12	12	12	12	12	12	12	12
Nepal	NP3	2020	12	12	12	12	12	12	12	12
Nepal	NP3	2021	12	12	12	12	12	12	12	12
Nepal	NP3	2022	12	12	12	12	12	12	12	12
Nepal	NP4	2019	12	12	12	12	12	12	12	12
Nepal	NP4	2020	12	12	12	12	12	12	12	12
Nepal	NP4	2021	12	12	12	12	12	12	12	12
Nepal	NP4	2022	12	12	12	12	12	12	12	12
Nigeria	NG1	2019	12	12	12	12	12	12	12	12
Nigeria	NG1	2020	12	12	12	12	12	12	12	12
Nigeria	NG1	2021	12	12	12	12	12	12	12	12
Nigeria	NG1	2022	12	12	12	12	12	12	12	12
Nigeria	NG2	2019	3	3	3	3	3	3	3	3
Nigeria	NG2	2020	12	12	12	12	12	12	12	12
Nigeria	NG2	2021	12	12	12	12	12	12	12	12
Nigeria	NG2	2022	12	12	12	12	12	12	12	12
Nigeria	NG3	2019	12	12	12	12	12	12	12	12
Nigeria	NG3	2020	12	12	12	12	12	12	12	12
Nigeria	NG3	2021	12	12	12	12	12	12	12	12
Nigeria	NG3	2022	12	12	12	12	12	12	12	12
Nigeria	NG4	2019	12	12	12	12	12	12	12	12
Nigeria	NG4	2020	12	12	12	12	12	12	12	12
Nigeria	NG4	2021	12	12	12	12	12	12	12	12
Nigeria	NG4	2022	12	12	12	12	12	12	12	12
Tanzania	TZ1	2019	12	12	12	0	0	0	0	12
Tanzania	TZ1	2020	12	12	12	0	0	0	0	12
Tanzania	TZ1	2021	12	12	12	0	0	0	0	12
Tanzania	TZ1	2022	12	12	12	0	0	0	0	12
Tanzania	TZ2	2019	12	12	12	12	12	12	12	12
Tanzania	TZ2	2020	12	12	12	12	12	12	12	12
Tanzania	TZ2	2021	12	12	12	12	12	12	12	12
Tanzania	TZ2	2022	12	12	12	12	12	12	12	12
Tanzania	TZ3	2019	12	12	11	1	1	1	1	12
Tanzania	TZ3	2020	12	12	12	1	1	1	1	12
Tanzania	TZ3	2021	12	12	11	0	0	0	0	12
Tanzania	TZ3	2022	12	12	12	0	0	0	0	12
Tanzania	TZ4	2019	12	12	12	0	0	0	0	12

				N months reporting on outcome						
**Country**	**Clinic ID**	**Year**	**N months open***	**Neonatal deaths**	**Maternal deaths**	**Congenital malformations**	**Late preterm**	**Very preterm**	**Extremely preterm**	**Stillbirth**
Bangladesh	BD1	2019	12	12	12	12	12	12	12	12
Bangladesh	BD1	2020	12	12	12	12	12	12	12	12
Tanzania	TZ4	2020	12	12	12	0	0	0	0	12
Tanzania	TZ4	2021	12	12	12	0	0	0	0	11
Tanzania	TZ4	2022	12	12	12	0	0	0	0	12
Tanzania	TZ5	2019	12	12	12	12	0	12	12	12
Tanzania	TZ5	2020	12	12	12	12	0	12	12	12
Tanzania	TZ5	2021	12	12	12	12	0	12	12	12
Tanzania	TZ5	2022	12	12	12	12	0	12	12	12
Uganda	UG1	2019	12	12	12	12	12	12	12	12
Uganda	UG1	2020	12	12	12	12	12	12	12	12
Uganda	UG1	2021	12	12	12	12	12	12	12	12
Uganda	UG1	2022	12	12	12	12	12	12	12	12
Uganda	UG2	2019	12	5	5	5	5	5	5	5
Uganda	UG2	2020	3	3	3	3	3	3	3	3
Uganda	UG2	2021	11	7	7	7	7	7	7	7
Uganda	UG2	2022	12	11	11	11	11	11	11	11
Uganda	UG3	2019	12	12	12	12	12	12	12	12
Uganda	UG3	2020	12	12	12	12	12	12	12	12
Uganda	UG3	2021	12	12	12	12	12	12	12	12
Uganda	UG3	2022	12	12	12	12	12	12	12	12
Uganda	UG4	2019	12	9	9	9	9	9	9	9
Uganda	UG4	2020	12	12	12	12	12	12	12	12
Uganda	UG4	2021	12	12	12	12	12	12	12	12
Uganda	UG4	2022	12	12	12	12	12	12	12	12

* Inferred by the availability of data for ANC visit counts, delivery counts, or pregnancy outcome counts.

Supplementary Table S3. AESI by clinic and year

Country	Clinic ID	Year	Neonatal deaths						Maternal deaths						Congenital malformations						Preterm (combined)					
			N deliveries	n outcomes	% outcomes	lower 95% CI, % outcomes	upper 95% CI, % outcomes	Country reference rate (%)	N deliveries	n outcomes	% outcomes	lower 95% CI, % outcomes	upper 95% CI, % outcomes	Country reference rate (%)	N deliveries	n outcomes	% outcomes	lower 95% CI, % outcomes	upper 95% CI, % outcomes	Country reference rate (%)	N deliveries	n outcomes	% outcomes	lower 95% CI, % outcomes	upper 95% CI, % outcomes	Country reference rate (%)
**Rates by country**																										
Bangladesh	–	2019–2022	35877	0	0	0	0.011	1.7	35877	0	0	0	0.011	0.123	35875	5	0.014	0.006	0.033	NA	35879	1749	4.875	4.657	5.103	16.2
Gambia	–	2019–2022	72996	420	0.575	0.523	0.633	2.4	72996	73	0.1	0.08	0.126	0.458	6955	0	0	0	0.055	NA	6955	0	0	0	0.055	14
Nepal	–	2019–2022	153515	127	0.734	0.693	0.778	1.7	153515	125	0.081	0.068	0.097	0.174	153515	729	0.475	0.442	0.511	NA	153515	9391	6.117	5.999	6.238	11.2
Nigeria	–	2019–2022	21816	430	1.971	1.795	2.164	3.4	21816	31	0.142	0.1	0.202	0.047	21816	123	0.564	0.473	0.672	NA	21816	2896	13.275	12.831	13.731	9.9
Tanza	–	2019–	34420	12	0.035	0.02	0.061	2	34234	6	0.018	0.008	0.038	0.238	12667	3	0.024	0.008	0.07	NA	3204	0	0	0	0.12	8.4

Country	Clinic ID	Year	Late preterm						Very preterm						Extremely preterm						Stillbirth					
			N deliveries	n outcomes	% outcomes	lower 95% CI, % outcomes	upper 95% CI, % outcomes	Country reference rate (%)	N deliveries	n outcomes	% outcomes	lower 95% CI, % outcomes	upper 95% CI, % outcomes	Country reference rate (%)	N deliveries	n outcomes	% outcomes	lower 95% CI, % outcomes	upper 95% CI, % outcomes	Country reference rate (%)	N deliveries	n outcomes	% outcomes	lower 95% CI, % outcomes	upper 95% CI, % outcomes	Country reference rate (%)
**Rates by country**																										
Bangladesh	–	2019–2022	35877	1474	4.108	3.908	4.319	NA	35877	157	0.438	0.374	0.511	NA	35877	118	0.329	0.275	0.394	NA	35877	126	0.351	0.295	0.418	2.05
Gambia	–	2019–2022	6955	0	0	0	0.055	NA	6955	0	0	0	0.055	NA	6955	0	0	0	0.055	NA	75359	1781	2.363	2.257	2.474	2.103
Nepal	–	2019–2022	153515	8122	5.291	5.18	5.404	NA	153515	760	0.495	0.461	0.531	NA	153515	509	0.332	0.304	0.362	NA	153515	2003	1.305	1.249	1.363	1.572
Nigeria	–	2019–2022	21816	2545	11.666	11.247	12.098	NA	21816	312	1.43	1.281	1.596	NA	21816	39	0.179	0.131	0.244	NA	21816	871	3.992	3.741	4.26	2.249
Tanza	–	2019–	3204	0	0	0	0.12	NA	12667	0	0	0	0.03	NA	12667	0	0	0	0.03	NA	34186	58	0.17	0.131	0.219	1.828

nia		2022																																																
Uganda	–	2019–2022	59141	112	0.189	0.157	0.228	1.8	59141	16	0.027	0.017	0.044	0.284	59141	38	0.064	0.047	0.088	NA	59141	3259	5.51	5.33	5.697	10	59141	3039	5.139	4.964	5.319	NA	59141	161	0.272	0.233	0.318	NA	59141	59	0.1	0.077	0.129	NA	59141	289	0.489	0.436	0.548	1.51
**Rates by clinic ID**																																																		
Bangladesh	BD1	2019–2022	25218	0	0	0	0.015	1.7	25218	0	0	0	0.015	0.123	25218	1	0.004	0	0.022	NA	25218	931	3.692	3.466	3.932	16.2	25218	677	2.685	2.492	2.891	NA	25218	137	0.543	0.46	0.642	NA	25218	117	0.464	0.387	0.556	NA	25218	2	0.008	0.002	0.029	2.05
Bangladesh	BD2	2019–2022	1774	0	0	0	0.216	1.7	1774	0	0	0	0.216	0.123	1774	0	0	0	0.216	NA	1774	45	2.537	1.901	3.377	16.2	1774	44	2.48	1.853	3.313	NA	1774	1	0.056	0.003	0.319	NA	1774	0	0	0	0.216	NA	1774	0	0	0	0.216	2.05
Bangladesh	BD3	2019–2022	3260	0	0	0	0.118	1.7	3260	0	0	0	0.118	0.123	3260	3	0.092	0.031	0.27	NA	3260	137	4.206	3.566	4.947	16.2	3260	135	4.141	3.509	4.881	NA	3260	2	0.061	0.017	0.223	NA	3260	0	0	0	0.118	NA	3260	67	2.055	1.622	2.602	2.05
Bangladesh	BD4	2019–2022	2239	0	0	0	0.171	1.7	2239	0	0	0	0.171	0.123	2239	0	0	0	0.171	NA	2239	45	2.01	1.505	2.679	16.2	2239	41	1.831	1.353	2.475	NA	2239	4	0.179	0.069	0.458	NA	2239	0	0	0	0.171	NA	2239	37	1.653	1.201	2.295	2.05
Bangladesh	BD5	2019–2022	3386	0	0	0	0.113	1.7	3386	0	0	0	0.113	0.123	3386	1	0.03	0.002	0.167	NA	3386	591	17.454	16.213	18.769	16.2	3386	577	17.041	15.812	18.344	NA	3386	13	0.384	0.225	0.656	NA	3386	1	0.03	0.002	0.167	NA	3386	20	0.591	0.383	0.911	2.05
Gambia	GM1	2019–2022	1193	0	0	0	0.321	2.4	1193	0	0	0	0.321	0.458	0	0	NA	NA	NA	NA	0	0	NA	NA	NA	14	0	0	NA	NA	NA	NA	0	0	NA	NA	NA	NA	0	0	NA	NA	NA	NA	3556	64	1.8	1.412	2.292	2.103

Gambia	GM2	2019–2022	30947	172	0.556	0.479	0.645	2.4	30947	26	0.084	0.057	0.123	0.458	6955	0	0	0	0.055	NA	6955	0	0	0	0.055	14	6955	0	0	0	0.055	NA	6955	0	0	0	0.055	NA	6955	0	0	0	0.055	NA	30947	801	2.588	2.417	2.771	2.103
Gambia	GM3	2019–2022	19629	172	0.876	0.755	1.017	2.4	19629	11	0.056	0.031	0.1	0.458	0	0	NA	NA	NA	NA	0	0	NA	NA	NA	14	0	0	NA	NA	NA	NA	0	0	NA	NA	NA	NA	0	0	NA	NA	NA	NA	19629	238	1.212	1.069	1.375	2.103
Gambia	GM4	2019–2022	11638	39	0.335	0.245	0.458	2.4	11638	30	0.258	0.181	0.368	0.458	0	0	NA	NA	NA	NA	0	0	NA	NA	NA	14	0	0	NA	NA	NA	NA	0	0	NA	NA	NA	NA	0	0	NA	NA	NA	NA	11638	480	4.124	3.778	4.501	2.103
Gambia	GM5	2019–2022	9589	37	0.386	0.28	0.531	2.4	9589	6	0.063	0.029	0.136	0.458	0	0	NA	NA	NA	NA	0	0	NA	NA	NA	14	0	0	NA	NA	NA	NA	0	0	NA	NA	NA	NA	0	0	NA	NA	NA	NA	9589	198	2.065	1.799	2.369	2.103
Nepal	NP1	2019–2022	20072	195	0.972	0.845	1.117	1.7	20072	33	0.164	0.117	0.231	0.174	20072	70	0.349	0.276	0.44	NA	20072	1941	9.67	9.269	10.087	11.2	20072	1594	7.941	7.575	8.324	NA	20072	166	0.827	0.711	0.962	NA	20072	181	0.902	0.78	1.042	NA	20072	436	2.172	1.979	2.383	1.572
Nepal	NP2	2019–2022	35102	117	0.333	0.278	0.399	1.7	35102	66	0.188	0.148	0.239	0.174	35102	150	0.427	0.364	0.501	NA	35102	2587	7.37	7.101	7.648	11.2	35102	2283	6.504	6.251	6.767	NA	35102	246	0.701	0.619	0.794	NA	35102	58	0.165	0.128	0.214	NA	35102	454	1.293	1.18	1.417	1.572
Nepal	NP3	2019–2022	3719	9	0.242	0.127	0.459	1.7	3719	2	0.054	0.015	0.196	0.174	3719	3	0.081	0.027	0.237	NA	3719	65	1.748	1.374	2.221	11.2	3719	52	1.398	1.068	1.829	NA	3719	6	0.161	0.074	0.352	NA	3719	7	0.188	0.091	0.388	NA	3719	34	0.914	0.655	1.275	1.572
Nepal	NP4	2019–2	94622	806	0.852	0.795	0.912	1.7	94622	24	0.025	0.017	0.038	0.174	94622	506	0.535	0.49	0.583	NA	94622	4798	5.071	4.933	5.212	11.2	94622	4193	4.431	4.302	4.564	NA	94622	342	0.361	0.325	0.402	NA	94622	263	0.278	0.246	0.314	NA	94622	1079	1.14	1.075	1.21	1.572

		022																																																
Nigeria	NG1	2019–2022	7698	126	1.637	1.377	1.945	3.4	7698	21	0.273	0.179	0.417	1.047	7698	101	1.312	1.081	1.592	NA	7698	773	10.042	9.39	10.733	9.9	7698	594	7.716	7.141	8.334	NA	7698	178	2.312	2	2.673	NA	7698	1	0.013	0.001	0.074	NA	7698	377	4.897	4.437	5.402	2.249
Nigeria	NG2	2019–2022	2465	48	1.947	1.472	2.572	3.4	2465	0	0	0	0.156	1.047	2465	1	0.041	0.002	0.229	NA	2465	363	14.726	13.382	16.18	9.9	2465	328	13.306	12.02	14.704	NA	2465	18	0.73	0.462	1.151	NA	2465	17	0.69	0.431	1.102	NA	2465	54	2.191	1.683	2.847	2.249
Nigeria	NG3	2019–2022	8251	64	0.776	0.608	0.989	3.4	8251	5	0.061	0.026	0.142	1.047	8251	5	0.061	0.026	0.142	NA	8251	1203	14.58	13.835	15.358	9.9	8251	1100	13.332	12.615	14.082	NA	8251	89	1.079	0.877	1.325	NA	8251	14	0.17	0.101	0.285	NA	8251	282	3.418	3.047	3.832	2.249
Nigeria	NG4	2019–2022	3402	192	5.644	4.917	6.47	3.4	3402	5	0.147	0.063	0.344	1.047	3402	16	0.47	0.29	0.763	NA	3402	557	16.373	15.167	17.654	9.9	3402	523	15.373	14.2	16.624	NA	3402	27	0.794	0.546	1.152	NA	3402	7	0.206	0.1	0.424	NA	3402	158	4.644	3.987	5.404	2.249
Tanzania	TZ1	2019–2022	8182	0	0	0	0.047	2	8182	0	0	0	0.047	0.238	0	0	NA	NA	NA	NA	0	0	NA	NA	NA	8.4	0	0	NA	NA	NA	NA	0	0	NA	NA	NA	NA	0	0	NA	NA	NA	NA	8182	0	0	0	0.047	1.828
Tanzania	TZ2	2019–2022	2952	5	0.169	0.072	0.396	2	2952	0	0	0	0.13	0.238	2952	0	0	0	0.13	NA	2952	0	0	0	0.13	8.4	2952	0	0	0	0.13	NA	2952	0	0	0	0.13	NA	2952	0	0	0	0.13	NA	2952	16	0.542	0.334	0.879	1.828
Tanzania	TZ3	2019–2022	5763	0	0	0	0.067	2	5577	0	0	0	0.069	0.238	252	0	0	0	1.501	NA	252	0	0	0	1.501	8.4	252	0	0	0	1.501	NA	252	0	0	0	1.501	NA	252	0	0	0	1.501	NA	5763	0	0	0	0.067	1.828
Tan	TZ4	201	80	0	0	0	0.0	2	80	2	0.0	0.0	0.	0.2	0	0	NA	NA	NA	NA	0	0	NA	NA	NA	8.4	0	0	NA	NA	NA	NA	0	0	NA	NA	NA	NA	0	0	NA	NA	NA	NA	78	16	0.2	0.1	0.3	1.8

zania		9–2022	60				48		60		25	07	09	38																															26		04	26	32	28
Tanzania	TZ5	2019–2022	9463	7	0.074	0.036	0.153	2	9463	4	0.042	0.016	0.109	0.238	9463	3	0.032	0.011	0.093	NA	0	0	NA	NA	NA	8.4	0	0	NA	NA	NA	NA	9463	0	0	0	0.041	NA	9463	0	0	0	0.041	NA	9463	26	0.275	0.188	0.402	1.828
Uganda	UG1	2019–2022	1020	0	0	0	0.375	1.8	1020	0	0	0	0.375	0.284	1020	0	0	0	0.375	NA	1020	16	1.569	0.968	2.533	10	1020	15	1.471	0.893	2.412	NA	1020	1	0.098	0.005	0.553	NA	1020	0	0	0	0.375	NA	1020	6	0.588	0.27	1.277	1.51
Uganda	UG2	2019–2022	6396	26	0.407	0.278	0.595	1.8	6396	3	0.047	0.016	0.138	0.284	6396	3	0.047	0.016	0.138	NA	6396	470	7.348	6.734	8.014	10	6396	426	6.66	6.075	7.298	NA	6396	31	0.485	0.342	0.687	NA	6396	13	0.203	0.119	0.347	NA	6396	60	0.938	0.73	1.206	1.51
Uganda	UG3	2019–2022	25138	83	0.33	0.266	0.409	1.8	25138	13	0.052	0.03	0.088	0.284	25138	23	0.091	0.061	0.137	NA	25138	1277	5.08	4.815	5.358	10	25138	1219	4.849	4.591	5.122	NA	25138	56	0.223	0.172	0.289	NA	25138	2	0.008	0.002	0.029	NA	25138	139	0.553	0.469	0.652	1.51
Uganda	UG4	2019–2022	26587	3	0.011	0.004	0.033	1.8	26587	0	0	0	0.014	0.284	26587	12	0.045	0.026	0.079	NA	26587	1496	5.627	5.356	5.91	10	26587	1379	5.187	4.927	5.46	NA	26587	73	0.275	0.218	0.345	NA	26587	44	0.165	0.123	0.222	NA	26587	84	0.316	0.255	0.391	1.51
**Rates by country and year**																																																		
Bangladesh	–	2019	7745	0	0	0	0.05	1.7	7745	0	0	0	0.05	0.123	7745	3	0.039	0.013	0.114	NA	7745	279	3.602	3.21	4.041	16.2	7745	275	3.551	3.161	3.986	NA	7745	0	0	0	0.05	NA	7745	4	0.052	0.02	0.133	NA	7745	29	0.374	0.261	0.537	2.05
Bangladesh	–	2020	8496	0	0	0	0.045	1.7	8496	0	0	0	0.045	0.123	8496	0	0	0	0.045	NA	8496	446	5.25	4.795	5.754	16.2	8496	356	4.19	3.784	4.637	NA	8496	6	0.071	0.032	0.154	NA	8496	84	0.989	0.799	1.222	NA	8496	28	0.33	0.228	0.476	2.05

Bangladesh	–	2021	9403	0	0	0	0.041	1.7	9403	0	0	0	0.041	0.123	9403	1	0.011	0.001	0.06	NA	9403	494	5.254	4.821	5.723	16.2	9403	406	4.318	3.925	4.748	NA	9403	58	0.617	0.477	0.796	NA	9403	30	0.319	0.224	0.455	NA	9403	28	0.298	0.206	0.43	2.05
Bangladesh	–	2022	10233	0	0	0	0.038	1.7	10233	0	0	0	0.038	0.123	10233	1	0.01	0.001	0.055	NA	10233	530	5.179	4.767	5.626	16.2	10233	437	4.27	3.896	4.68	NA	10233	93	0.909	0.742	1.112	NA	10233	0	0	0	0.038	NA	10233	41	0.401	0.295	0.543	2.05
Gambia	–	2019	16243	227	1.398	1.228	1.59	2.4	16243	25	0.154	0.104	0.227	0.458	0	0	NA	NA	NA	NA	0	0	NA	NA	NA	14	0	0	NA	NA	NA	NA	0	0	NA	NA	NA	NA	0	0	NA	NA	NA	NA	17677	436	2.466	2.248	2.706	2.103
Gambia	–	2020	18853	55	0.292	0.224	0.379	2.4	18853	17	0.09	0.056	0.144	0.458	6955	0	0	0	0.055	NA	6955	0	0	0	0.055	14	6955	0	0	0	0.055	NA	6955	0	0	0	0.055	NA	6955	0	0	0	0.055	NA	19782	484	2.447	2.24	2.671	2.103
Gambia	–	2021	18168	65	0.358	0.281	0.456	2.4	18168	13	0.072	0.042	0.122	0.458	0	0	NA	NA	NA	NA	0	0	NA	NA	NA	14	0	0	NA	NA	NA	NA	0	0	NA	NA	NA	NA	0	0	NA	NA	NA	NA	18168	437	2.405	2.192	2.638	2.103
Gambia	–	2022	19732	73	0.37	0.294	0.465	2.4	19732	18	0.091	0.058	0.144	0.458	0	0	NA	NA	NA	NA	0	0	NA	NA	NA	14	0	0	NA	NA	NA	NA	0	0	NA	NA	NA	NA	0	0	NA	NA	NA	NA	19732	424	2.149	1.956	2.361	2.103
Nepal	–	2019	41189	229	0.556	0.489	0.633	1.7	41189	24	0.058	0.039	0.087	0.174	41189	167	0.405	0.349	0.472	NA	41189	2437	5.917	5.693	6.149	11.2	41189	2091	5.077	4.869	5.293	NA	41189	177	0.43	0.371	0.498	NA	41189	169	0.41	0.353	0.477	NA	41189	524	1.272	1.168	1.385	1.572
Nepal	–	2020	37406	350	0.936	0.843	1.038	1.7	37406	54	0.144	0.111	0.188	0.174	37406	176	0.471	0.406	0.545	NA	37406	2103	5.622	5.393	5.86	11.2	37406	1793	4.793	4.581	5.015	NA	37406	203	0.543	0.473	0.622	NA	37406	107	0.286	0.237	0.346	NA	37406	494	1.321	1.21	1.441	1.572
Nepal	–	2021	36786	312	0.848	0.759	0.947	1.7	36786	30	0.082	0.057	0.116	0.174	36786	183	0.497	0.431	0.575	NA	36786	2485	6.755	6.503	7.016	11.2	36786	2159	5.869	5.633	6.114	NA	36786	193	0.525	0.456	0.604	NA	36786	133	0.362	0.305	0.428	NA	36786	509	1.384	1.269	1.508	1.572
Nepal	–	2022	38134	236	0.619	0.545	0.703	1.7	38134	17	0.045	0.028	0.071	0.174	38134	203	0.532	0.464	0.611	NA	38134	2366	6.204	5.967	6.451	11.2	38134	2079	5.452	5.228	5.684	NA	38134	187	0.49	0.425	0.566	NA	38134	100	0.262	0.216	0.319	NA	38134	476	1.248	1.142	1.365	1.572
Nigeria	–	2019	6068	85	1.401	1.134	1.729	3.4	6068	7	0.115	0.056	0.238	1.047	6068	27	0.445	0.306	0.647	NA	6068	865	14.255	13.398	15.157	9.9	6068	781	12.871	12.052	13.737	NA	6068	80	1.318	1.061	1.638	NA	6068	4	0.066	0.026	0.169	NA	6068	242	3.988	3.524	4.51	2.249
Nigeria	–	2020	5383	56	1.04	0.802	1.348	3.4	5383	8	0.149	0.075	0.293	1.047	5383	40	0.743	0.546	1.01	NA	5383	665	12.354	11.501	13.26	9.9	5383	597	11.09	10.279	11.957	NA	5383	58	1.077	0.834	1.39	NA	5383	10	0.186	0.101	0.342	NA	5383	250	4.644	4.114	5.239	2.249

Nigeria	–	2021	5729	138	2.409	2.043	2.839	3.4	5729	8	0.14	0.071	0.275	1.047	5729	33	0.576	0.41	0.808	NA	5729	704	12.288	11.463	13.164	9.9	5729	613	10.7	9.926	11.527	NA	5729	77	1.344	1.077	1.676	NA	5729	14	0.244	0.146	0.41	NA	5729	193	3.369	2.932	3.868	2.249
Nigeria	–	2022	4636	151	3.257	2.784	3.808	3.4	4636	8	0.173	0.087	0.34	1.047	4636	23	0.496	0.331	0.743	NA	4636	662	14.28	13.302	15.316	9.9	4636	554	11.95	11.048	12.915	NA	4636	97	2.092	1.718	2.546	NA	4636	11	0.237	0.133	0.424	NA	4636	186	4.012	3.484	4.616	2.249
Tanzania	–	2019	6161	1	0.016	0.001	0.092	2	6075	1	0.016	0.001	0.093	0.238	2387	0	0	0	0.161	NA	234	0	0	0	1.615	8.4	234	0	0	0	1.615	NA	2387	0	0	0	0.161	NA	2387	0	0	0	0.161	NA	6161	15	0.243	0.148	0.401	1.828
Tanzania	–	2020	7962	2	0.025	0.007	0.092	2	7962	0	0	0	0.048	0.238	3070	1	0.033	0.002	0.184	NA	928	0	0	0	0.412	8.4	928	0	0	0	0.412	NA	3070	0	0	0	0.125	NA	3070	0	0	0	0.125	NA	7962	19	0.239	0.153	0.372	1.828
Tanzania	–	2021	9760	5	0.051	0.012	0.12	2	9660	1	0.01	0.001	0.059	0.237	3577	2	0.056	0.015	0.204	NA	1084	0	0	0	0.353	8.4	1084	0	0	0	0.353	NA	3577	0	0	0	0.107	NA	3577	0	0	0	0.107	NA	9526	11	0.115	0.064	0.207	1.828
Tanzania	–	2022	10537	4	0.038	0.015	0.098	2	10537	4	0.038	0.015	0.098	0.238	3633	0	0	0	0.106	NA	958	0	0	0	0.399	8.4	958	0	0	0	0.399	NA	3633	0	0	0	0.106	NA	3633	0	0	0	0.106	NA	10537	13	0.123	0.072	0.211	1.828
Uganda	–	2019	13405	31	0.231	0.163	0.328	1.8	13405	4	0.03	0.012	0.077	0.284	13405	7	0.052	0.025	0.108	NA	13405	805	6.005	5.615	6.42	10	13405	758	5.655	5.276	6.058	NA	13405	36	0.269	0.194	0.372	NA	13405	11	0.082	0.046	0.147	NA	13405	71	0.53	0.42	0.667	1.51
Uganda	–	2020	15354	25	0.163	0.11	0.24	1.8	15354	3	0.02	0.007	0.057	0.284	15354	10	0.065	0.035	0.12	NA	15354	768	5.002	4.668	5.358	10	15354	707	4.605	4.284	4.948	NA	15354	46	0.3	0.225	0.399	NA	15354	15	0.098	0.059	0.161	NA	15354	68	0.443	0.35	0.561	1.51
Uganda	–	2021	13287	23	0.173	0.115	0.26	1.8	13287	4	0.03	0.012	0.077	0.284	13287	7	0.053	0.026	0.109	NA	13287	681	5.125	4.763	5.513	10	13287	635	4.779	4.429	5.155	NA	13287	34	0.256	0.183	0.357	NA	13287	12	0.09	0.052	0.158	NA	13287	67	0.504	0.397	0.64	1.51
Uganda	–	2022	17095	33	0.193	0.137	0.271	1.8	17095	5	0.029	0.012	0.068	0.284	17095	14	0.082	0.049	0.137	NA	17095	1005	5.879	5.536	6.242	10	17095	939	5.493	5.161	5.844	NA	17095	45	0.263	0.197	0.352	NA	17095	21	0.123	0.08	0.188	NA	17095	83	0.486	0.392	0.601	1.51
**Rates by clinic ID and year**																																																		
Bangladesh	BD1	2019	5496	0	0	0	0.07	1.7	5496	0	0	0	0.07	0.123	5496	1	0.018	0.001	0.103	NA	5496	132	2.402	2.029	2.841	16.2	5496	128	2.329	1.962	2.762	NA	5496	0	0	0	0.07	NA	5496	4	0.073	0.028	0.187	NA	5496	1	0.018	0.001	0.103	2.05

Bangladesh	BD1	2020	6088	0	0	0	0.063	1.7	6088	0	0	0	0.063	0.123	6088	0	0	0	0.063	NA	6088	301	4.944	4.427	5.518	16.2	6088	217	3.564	3.127	4.06	NA	6088	0	0	0	0.063	NA	6088	84	1.38	1.116	1.705	NA	6088	1	0.016	0.001	0.093	2.05
Bangladesh	BD1	2021	6622	0	0	0	0.058	1.7	6622	0	0	0	0.058	0.123	6622	0	0	0	0.058	NA	6622	278	4.198	3.741	4.708	16.2	6622	193	2.915	2.536	3.348	NA	6622	56	0.846	0.652	1.096	NA	6622	29	0.438	0.305	0.628	NA	6622	0	0	0	0.058	2.05
Bangladesh	BD1	2022	7012	0	0	0	0.055	1.7	7012	0	0	0	0.055	0.123	7012	0	0	0	0.055	NA	7012	220	3.137	2.754	3.572	16.2	7012	139	1.982	1.681	2.336	NA	7012	81	1.155	0.93	1.433	NA	7012	0	0	0	0.055	NA	7012	0	0	0	0.055	2.05
Bangladesh	BD2	2019	387	0	0	0	0.983	1.7	387	0	0	0	0.983	0.123	387	0	0	0	0.983	NA	387	14	3.618	2.167	5.98	16.2	387	14	3.618	2.167	5.98	NA	387	0	0	0	0.983	NA	387	0	0	0	0.983	NA	387	0	0	0	0.983	2.05
Bangladesh	BD2	2020	254	0	0	0	1.49	1.7	254	0	0	0	1.49	0.123	254	0	0	0	1.49	NA	254	10	3.937	2.152	7.094	16.2	254	10	3.937	2.152	7.094	NA	254	0	0	0	1.49	NA	254	0	0	0	1.49	NA	254	0	0	0	1.49	2.05
Bangladesh	BD2	2021	462	0	0	0	0.825	1.7	462	0	0	0	0.825	0.123	462	0	0	0	0.825	NA	462	9	1.948	1.028	3.66	16.2	462	9	1.948	1.028	3.66	NA	462	0	0	0	0.825	NA	462	0	0	0	0.825	NA	462	0	0	0	0.825	2.05
Bangladesh	BD2	2022	671	0	0	0	0.569	1.7	671	0	0	0	0.569	0.123	671	0	0	0	0.569	NA	671	12	1.788	1.026	3.1	16.2	671	11	1.639	0.918	2.91	NA	671	1	0.149	0.008	0.839	NA	671	0	0	0	0.569	NA	671	0	0	0	0.569	2.05
Bangladesh	BD3	2019	832	0	0	0	0.46	1.7	832	0	0	0	0.46	0.123	832	2	0.24	0.066	0.872	NA	832	74	8.894	7.144	11.022	16.2	832	74	8.894	7.144	11.022	NA	832	0	0	0	0.46	NA	832	0	0	0	0.46	NA	832	15	1.803	1.096	2.953	2.05
Bangla	BD3	2020	735	0	0	0	0.52	1.7	735	0	0	0	0.52	0.123	735	0	0	0	0.52	NA	735	13	1.769	1.037	3.002	16.2	735	11	1.497	0.838	2.66	NA	735	2	0.272	0.075	0.987	NA	735	0	0	0	0.52	NA	735	18	2.449	1.555	3.838	2.05

desh																																																		
Bangladesh	BD3	2021	831	0	0	0	0.46	1.7	831	0	0	0	0.46	0.123	831	0	0	0	0.46	NA	831	41	4.934	3.658	6.625	16.2	831	41	4.934	3.658	6.625	NA	831	0	0	0	0.46	NA	831	0	0	0	0.46	NA	831	12	1.444	0.828	2.507	2.05
Bangladesh	BD3	2022	862	0	0	0	0.444	1.7	862	0	0	0	0.444	0.123	862	1	0.116	0.006	0.654	NA	862	9	1.044	0.55	1.972	16.2	862	9	1.044	0.55	1.972	NA	862	0	0	0	0.444	NA	862	0	0	0	0.444	NA	862	22	2.552	1.691	3.834	2.05
Bangladesh	BD4	2019	383	0	0	0	0.993	1.7	383	0	0	0	0.993	0.123	383	0	0	0	0.993	NA	383	7	1.828	0.888	3.724	16.2	383	7	1.828	0.888	3.724	NA	383	0	0	0	0.993	NA	383	0	0	0	0.993	NA	383	12	3.133	1.801	5.396	2.05
Bangladesh	BD4	2020	592	0	0	0	0.645	1.7	592	0	0	0	0.645	0.123	592	0	0	0	0.645	NA	592	11	1.858	1.041	3.296	16.2	592	9	1.52	0.802	2.864	NA	592	2	0.338	0.093	1.223	NA	592	0	0	0	0.645	NA	592	9	1.52	0.802	2.864	2.05
Bangladesh	BD4	2021	563	0	0	0	0.678	1.7	563	0	0	0	0.678	0.123	563	0	0	0	0.678	NA	563	10	1.776	0.968	3.238	16.2	563	10	1.776	0.968	3.238	NA	563	0	0	0	0.678	NA	563	0	0	0	0.678	NA	563	8	1.421	0.722	2.779	2.05
Bangladesh	BD4	2022	701	0	0	0	0.545	1.7	701	0	0	0	0.545	0.123	701	0	0	0	0.545	NA	701	17	2.425	1.52	3.849	16.2	701	15	2.14	1.301	3.5	NA	701	2	0.285	0.078	1.034	NA	701	0	0	0	0.545	NA	701	8	1.141	0.579	2.236	2.05
Bangladesh	BD5	2019	647	0	0	0	0.59	1.7	647	0	0	0	0.59	0.123	647	0	0	0	0.59	NA	647	52	8.037	6.181	10.388	16.2	647	52	8.037	6.181	10.388	NA	647	0	0	0	0.59	NA	647	0	0	0	0.59	NA	647	1	0.15	0.008	0.87	2.05
Bangladesh	BD5	2020	827	0	0	0	0.462	1.7	827	0	0	0	0.462	0.123	827	0	0	0	0.462	NA	827	111	13.422	11.267	15.915	16.2	827	109	13.18	11.044	15.657	NA	827	2	0.242	0.066	0.877	NA	827	0	0	0	0.462	NA	827	0	0	0	0.462	2.05

Bangladesh	BD5	2021	925	0	0	0	0.414	1.7	925	0	0	0	0.414	0.123	925	1	0.108	0.006	0.61	NA	925	156	16.865	14.59	19.414	16.2	925	153	16.541	14.286	19.072	NA	925	2	0.216	0.059	0.785	NA	925	1	0.108	0.006	0.61	NA	925	8	0.865	0.439	1.697	2.05
Bangladesh	BD5	2022	987	0	0	0	0.388	1.7	987	0	0	0	0.388	0.123	987	0	0	0	0.388	NA	987	272	27.558	24.862	30.429	16.2	987	263	26.646	23.983	29.491	NA	987	9	0.912	0.48	1.724	NA	987	0	0	0	0.388	NA	987	11	1.114	0.623	1.985	2.05
Gambia	GM1	2019	0	0	NA	NA	NA	2.4	0	0	NA	NA	NA	0.458	0	0	NA	NA	NA	NA	0	0	NA	NA	NA	14	0	0	NA	NA	NA	NA	0	0	NA	NA	NA	NA	0	0	NA	NA	NA	NA	1434	32	2.232	1.585	3.133	2.103
Gambia	GM1	2020	0	0	NA	NA	NA	2.4	0	0	NA	NA	NA	0.458	0	0	NA	NA	NA	NA	0	0	NA	NA	NA	14	0	0	NA	NA	NA	NA	0	0	NA	NA	NA	NA	0	0	NA	NA	NA	NA	929	16	1.722	1.063	2.779	2.103
Gambia	GM1	2021	511	0	0	0	0.746	2.4	511	0	0	0	0.746	0.458	0	0	NA	NA	NA	NA	0	0	NA	NA	NA	14	0	0	NA	NA	NA	NA	0	0	NA	NA	NA	NA	0	0	NA	NA	NA	NA	511	7	1.37	0.665	2.8	2.103
Gambia	GM1	2022	682	0	0	0	0.56	2.4	682	0	0	0	0.56	0.458	0	0	NA	NA	NA	NA	0	0	NA	NA	NA	14	0	0	NA	NA	NA	NA	0	0	NA	NA	NA	NA	0	0	NA	NA	NA	NA	682	9	1.32	0.696	2.489	2.103
Gambia	GM2	2019	7395	161	2.179	1.869	2.535	2.4	7395	12	0.162	0.093	0.283	0.458	0	0	NA	NA	NA	NA	0	0	NA	NA	NA	14	0	0	NA	NA	NA	NA	0	0	NA	NA	NA	NA	0	0	NA	NA	NA	NA	7395	178	2.407	2.082	2.782	2.103
Gambia	GM2	2020	7700	6	0.078	0.036	0.17	2.4	7700	4	0.052	0.02	0.134	0.458	695	0	0	0	0.05	NA	695	0	0	0	0.05	14	695	0	0	0	0.05	NA	695	0	0	0	0.05	NA	695	0	0	0	0.05	NA	7700	190	2.468	2.144	2.838	2.103
Gambia	GM2	2021	7630	4	0.052	0.002	0.135	2.4	7630	5	0.066	0.028	0.153	0.458	0	0	NA	NA	NA	NA	0	0	NA	NA	NA	14	0	0	NA	NA	NA	NA	0	0	NA	NA	NA	NA	0	0	NA	NA	NA	NA	7630	220	2.883	2.531	3.283	2.103
Gambia	GM2	2022	8222	1	0.012	0.001	0.069	2.4	8222	5	0.061	0.026	0.142	0.458	0	0	NA	NA	NA	NA	0	0	NA	NA	NA	14	0	0	NA	NA	NA	NA	0	0	NA	NA	NA	NA	0	0	NA	NA	NA	NA	8222	213	2.591	2.269	2.957	2.103
Gambia	GM3	2019	4225	44	1.047	0.77	1.395	2.4	4225	1	0.024	0.001	0.134	0.458	0	0	NA	NA	NA	NA	0	0	NA	NA	NA	14	0	0	NA	NA	NA	NA	0	0	NA	NA	NA	NA	0	0	NA	NA	NA	NA	4225	61	1.444	1.126	1.85	2.103
Gambia	GM3	2020	4094	43	1.05	0.781	1.412	2.4	4094	5	0.122	0.052	0.286	0.458	0	0	NA	NA	NA	NA	0	0	NA	NA	NA	14	0	0	NA	NA	NA	NA	0	0	NA	NA	NA	NA	0	0	NA	NA	NA	NA	4094	54	1.319	1.012	1.717	2.103
Gam	GM3	20	57	40	0.7	0.5	0.9	2.4	57	0	0	0	0.0	0.4	0	0	NA	NA	NA	NA	0	0	NA	NA	NA	14	0	0	NA	NA	NA	NA	0	0	NA	NA	NA	NA	0	0	NA	NA	NA	NA	57	68	1.1	0.	1.5	2.1

bia		21	11			15	52		11				67	58																															11		91	94	07	03
Gambia	GM3	2022	5599	45	0.804	0.601	1.074	2.4	5599	5	0.089	0.038	0.209	0.458	0	0	NA	NA	NA	NA	0	0	NA	NA	NA	14	0	0	NA	NA	NA	NA	0	0	NA	NA	NA	NA	0	0	NA	NA	NA	NA	5599	55	0.982	0.756	1.276	2.103
Gambia	GM4	2019	2815	14	0.497	0.296	0.833	2.4	2815	9	0.32	0.168	0.607	0.458	0	0	NA	NA	NA	NA	0	0	NA	NA	NA	14	0	0	NA	NA	NA	NA	0	0	NA	NA	NA	NA	0	0	NA	NA	NA	NA	2815	114	4.05	3.382	4.843	2.103
Gambia	GM4	2020	2882	0	0	0	0.133	2.4	2882	6	0.208	0.095	0.453	0.458	0	0	NA	NA	NA	NA	0	0	NA	NA	NA	14	0	0	NA	NA	NA	NA	0	0	NA	NA	NA	NA	0	0	NA	NA	NA	NA	2882	142	4.927	4.195	5.779	2.103
Gambia	GM4	2021	2708	8	0.295	0.15	0.582	2.4	2708	7	0.258	0.125	0.533	0.458	0	0	NA	NA	NA	NA	0	0	NA	NA	NA	14	0	0	NA	NA	NA	NA	0	0	NA	NA	NA	NA	0	0	NA	NA	NA	NA	2708	114	4.21	3.516	5.033	2.103
Gambia	GM4	2022	3233	17	0.526	0.329	0.841	2.4	3233	8	0.247	0.125	0.488	0.458	0	0	NA	NA	NA	NA	0	0	NA	NA	NA	14	0	0	NA	NA	NA	NA	0	0	NA	NA	NA	NA	0	0	NA	NA	NA	NA	3233	110	3.402	2.831	4.085	2.103
Gambia	GM5	2019	1808	8	0.442	0.224	0.871	2.4	1808	3	0.166	0.056	0.487	0.458	0	0	NA	NA	NA	NA	0	0	NA	NA	NA	14	0	0	NA	NA	NA	NA	0	0	NA	NA	NA	NA	0	0	NA	NA	NA	NA	1808	51	2.821	2.152	3.69	2.103
Gambia	GM5	2020	4177	6	0.144	0.066	0.313	2.4	4177	2	0.048	0.013	0.174	0.458	0	0	NA	NA	NA	NA	0	0	NA	NA	NA	14	0	0	NA	NA	NA	NA	0	0	NA	NA	NA	NA	0	0	NA	NA	NA	NA	4177	82	1.963	1.584	2.43	2.103
Gambia	GM5	2021	1608	13	0.808	0.473	1.378	2.4	1608	1	0.062	0.003	0.351	0.458	0	0	NA	NA	NA	NA	0	0	NA	NA	NA	14	0	0	NA	NA	NA	NA	0	0	NA	NA	NA	NA	0	0	NA	NA	NA	NA	1608	28	1.741	1.207	2.505	2.103
Gambia	GM5	2022	1996	10	0.501	0.272	0.92	2.4	1996	0	0	0	0.192	0.458	0	0	NA	NA	NA	NA	0	0	NA	NA	NA	14	0	0	NA	NA	NA	NA	0	0	NA	NA	NA	NA	0	0	NA	NA	NA	NA	1996	37	1.854	1.348	2.545	2.103
Nepal	NP1	2019	5788	39	0.674	0.493	0.92	1.7	5788	7	0.121	0.059	0.249	0.174	5788	14	0.242	0.144	0.406	NA	5788	533	9.209	8.491	9.981	11.2	5788	452	7.809	7.146	8.529	NA	5788	36	0.622	0.45	0.86	NA	5788	45	0.777	0.582	1.039	NA	5788	99	1.71	1.407	2.078	1.572
Nepal	NP1	2020	4256	52	1.222	0.939	1.599	1.7	4256	12	0.282	0.161	0.492	0.174	4256	14	0.329	0.196	0.551	NA	4256	465	10.926	10.024	11.898	11.2	4256	376	8.835	8.019	9.725	NA	4256	44	1.057	0.791	1.412	NA	4256	44	1.034	0.771	1.385	NA	4256	105	2.467	2.042	2.978	1.572
Nepal	NP1	2021	4642	46	0.991	0.741	1.319	1.7	4642	9	0.194	0.102	0.368	0.174	4642	25	0.539	0.365	0.794	NA	4642	493	10.62	9.766	11.54	11.2	4642	387	8.337	7.576	9.167	NA	4642	50	1.077	0.818	1.417	NA	4642	56	1.206	0.93	1.563	NA	4642	118	2.542	2.127	3.036	1.572
Nepal	NP1	2022	5386	58	1.077	0.834	1.389	1.7	5386	5	0.093	0.04	0.217	0.174	5386	17	0.316	0.197	0.505	NA	5386	450	8.355	7.645	9.124	11.2	5386	379	7.037	6.384	7.751	NA	5386	35	0.65	0.468	0.902	NA	5386	36	0.668	0.483	0.924	NA	5386	114	2.117	1.765	2.537	1.572

Nepal	NP2	2019	11393	26	0.228	0.156	0.334	1.7	11393	10	0.088	0.048	0.162	0.174	11393	64	0.562	0.44	0.717	NA	11393	798	7.004	6.55	7.488	11.2	11393	702	6.162	5.735	6.618	NA	11393	73	0.641	0.51	0.805	NA	11393	23	0.202	0.135	0.303	NA	11393	122	1.071	0.898	1.277	1.572
Nepal	NP2	2020	8707	26	0.299	0.204	0.437	1.7	8707	35	0.402	0.289	0.559	0.174	8707	30	0.345	0.241	0.491	NA	8707	568	6.523	6.024	7.062	11.2	8707	490	5.628	5.163	6.132	NA	8707	69	0.792	0.627	1.002	NA	8707	9	0.103	0.054	0.196	NA	8707	132	1.516	1.28	1.795	1.572
Nepal	NP2	2021	8155	38	0.466	0.34	0.639	1.7	8155	11	0.135	0.075	0.241	0.175	8155	9	0.11	0.058	0.21	NA	8155	716	8.78	8.185	9.414	11.2	8155	652	7.995	7.426	8.604	NA	8155	52	0.638	0.487	0.835	NA	8155	12	0.147	0.084	0.257	NA	8155	114	1.398	1.165	1.677	1.572
Nepal	NP2	2022	6847	27	0.394	0.271	0.573	1.7	6847	10	0.146	0.079	0.269	0.174	6847	47	0.686	0.517	0.912	NA	6847	505	7.375	6.78	8.019	11.2	6847	439	6.412	5.855	7.017	NA	6847	52	0.759	0.58	0.994	NA	6847	14	0.204	0.122	0.343	NA	6847	86	1.256	1.018	1.548	1.572
Nepal	NP3	2019	905	0	0	0	0.423	1.7	905	0	0	0	0.423	0.174	905	2	0.221	0.061	0.802	NA	905	22	2.431	1.611	3.653	11.2	905	18	1.989	1.262	3.122	NA	905	2	0.221	0.061	0.802	NA	905	2	0.221	0.061	0.802	NA	905	3	0.331	0.113	0.97	1.572
Nepal	NP3	2020	1194	3	0.251	0.085	0.736	1.7	1194	2	0.168	0.046	0.609	0.174	1194	1	0.084	0.004	0.473	NA	1194	20	1.675	1.087	2.573	11.2	1194	17	1.424	0.891	2.268	NA	1194	2	0.168	0.046	0.609	NA	1194	1	0.084	0.004	0.473	NA	1194	16	1.34	0.827	2.162	1.572
Nepal	NP3	2021	896	4	0.446	0.174	1.142	1.7	896	0	0	0	0.427	0.174	896	0	0	0	0.427	NA	896	14	1.563	0.93	2.606	11.2	896	11	1.228	0.687	2.185	NA	896	1	0.112	0.006	0.629	NA	896	2	0.223	0.061	0.81	NA	896	7	0.781	0.379	1.604	1.572
Nepal	NP3	2022	724	2	0.276	0.076	1.002	1.7	724	0	0	0	0.528	0.174	724	0	0	0	0.528	NA	724	9	1.243	0.655	2.346	11.2	724	6	0.829	0.38	1.796	NA	724	1	0.138	0.007	0.778	NA	724	2	0.276	0.076	1.002	NA	724	8	1.105	0.561	2.165	1.572
Nepal	NP4	2019	23103	164	0.71	0.61	0.827	1.7	23103	7	0.03	0.015	0.063	0.174	23103	87	0.377	0.305	0.464	NA	23103	1084	4.692	4.427	4.972	11.2	23103	919	3.978	3.733	4.238	NA	23103	66	0.286	0.225	0.363	NA	23103	99	0.429	0.352	0.521	NA	23103	300	1.299	1.16	1.453	1.572
Nepal	NP4	2020	23249	269	1.157	1.027	1.303	1.7	23249	5	0.022	0.009	0.05	0.174	23249	131	0.563	0.475	0.668	NA	23249	1050	4.516	4.257	4.791	11.2	23249	910	3.914	3.672	4.171	NA	23249	87	0.374	0.304	0.461	NA	23249	53	0.228	0.174	0.298	NA	23249	241	1.037	0.914	1.175	1.572
Nepal	NP4	2021	23093	224	0.97	0.851	1.105	1.7	23093	10	0.043	0.024	0.08	0.174	23093	149	0.645	0.55	0.757	NA	23093	1262	5.465	5.179	5.765	11.2	23093	1109	4.802	4.534	5.086	NA	23093	90	0.39	0.317	0.479	NA	23093	63	0.273	0.213	0.349	NA	23093	270	1.169	1.038	1.316	1.572
Nepal	NP4	2022	25177	149	0.592	0.504	0.694	1.7	25177	2	0.008	0.002	0.029	0.174	25177	139	0.552	0.468	0.651	NA	25177	1402	5.569	5.292	5.859	11.2	25177	1255	4.985	4.723	5.26	NA	25177	99	0.393	0.323	0.478	NA	25177	48	0.191	0.144	0.253	NA	25177	268	1.064	0.945	1.199	1.572
Nigeria	NG1	2019	2395	20	0.835	0.541	1.286	3.4	2395	4	0.167	0.065	0.429	1.047	2395	20	0.835	0.541	1.286	NA	2395	174	7.265	6.293	8.375	9.9	2395	125	5.219	4.398	6.184	NA	2395	49	2.046	1.551	2.694	NA	2395	0	0	0	0.16	NA	2395	110	4.593	3.825	5.206	2.249
Nigeria	NG1	2020	2062	28	1.3	0.9	1.9	3.4	2062	6	0.2	0.1	0.6	1.0	2062	34	1.6	1.1	2.2	NA	2062	160	7.7	6.6	8.9	9.9	2062	119	5.7	4.8	6.8	NA	2062	41	1.9	1.4	2.6	NA	2062	0	0	0	0.1	NA	2062	116	5.6	4.7	6.7	2.2

					58	41	56				91	33	33	47			49	82	95				59	82	94				71	44	62				88	69	86						86				26	11	05	49
Nigeria	NG1	2021	1669	30	1.797	1.262	2.554	3.4	1669	5	0.3	0.128	0.699	1.047	1669	31	1.857	1.312	2.624	NA	1669	156	9.347	8.042	10.838	9.9	1669	121	7.25	6.102	8.595	NA	1669	35	2.097	1.512	2.902	NA	1669	0	0	0	0.23	NA	1669	71	4.254	3.386	5.332	2.249
Nigeria	NG1	2022	1572	48	3.053	2.311	4.025	3.4	1572	6	0.382	0.175	0.83	1.047	1572	16	1.018	0.627	1.647	NA	1572	283	18.003	16.182	19.979	9.9	1572	229	14.567	12.91	16.398	NA	1572	53	3.372	2.587	4.384	NA	1572	1	0.064	0.003	0.359	NA	1572	80	5.089	4.108	6.289	2.249
Nigeria	NG2	2019	25	0	0	0	13.319	3.4	25	0	0	0	13.319	10.47	25	0	0	0	13.319	NA	25	7	28	14.284	47.577	9.9	25	7	28	14.284	47.577	NA	25	0	0	0	13.319	NA	25	0	0	0	13.319	NA	25	0	0	0	13.319	2.249
Nigeria	NG2	2020	324	2	0.617	0.169	2.222	3.4	324	0	0	0	1.172	1.047	324	0	0	0	1.172	NA	324	64	19.753	15.783	24.432	9.9	324	58	17.901	14.111	22.444	NA	324	3	0.926	0.315	2.687	NA	324	3	0.926	0.315	2.687	NA	324	9	2.778	1.468	5.194	2.249
Nigeria	NG2	2021	1343	27	2.01	1.385	2.909	3.4	1343	0	0	0	0.285	1.047	1343	1	0.074	0.004	0.421	NA	1343	191	14.222	12.456	16.192	9.9	1343	176	13.105	11.405	15.016	NA	1343	6	0.447	0.205	0.971	NA	1343	9	0.67	0.353	1.269	NA	1343	32	2.383	1.693	3.344	2.249
Nigeria	NG2	2022	773	19	2.458	1.579	3.807	3.4	773	0	0	0	0.494	1.047	773	0	0	0	0.494	NA	773	101	13.066	10.872	15.626	9.9	773	87	11.255	9.216	13.677	NA	773	9	1.164	0.614	2.198	NA	773	5	0.647	0.277	1.505	NA	773	13	1.682	0.985	2.856	2.249
Nigeria	NG3	2019	2599	2	0.077	0.021	0.28	3.4	2599	2	0.077	0.021	0.28	1.047	2599	0	0	0	0.148	NA	2599	362	13.928	12.65	15.313	9.9	2599	335	12.89	11.656	14.233	NA	2599	25	0.962	0.652	1.416	NA	2599	2	0.077	0.021	0.28	NA	2599	88	3.386	2.756	4.153	2.249
Nigeria	NG3	2020	2199	1	0.045	0.002	0.257	3.4	2199	0	0	0	0.174	1.047	2199	4	0.182	0.071	0.467	NA	2199	345	15.689	14.229	17.269	9.9	2199	326	14.825	13.401	16.371	NA	2199	14	0.637	0.38	1.066	NA	2199	5	0.227	0.097	0.531	NA	2199	81	3.683	2.974	4.555	2.249
Nigeria	NG3	2021	1902	32	1.682	1.194	2.365	3.4	1902	1	0.053	0.003	0.297	1.047	1902	0	0	0	0.202	NA	1902	274	14.406	12.899	16.056	9.9	1902	249	13.091	11.65	14.682	NA	1902	22	1.157	0.765	1.745	NA	1902	3	0.158	0.054	0.463	NA	1902	58	3.049	2.366	3.922	2.249
Nigeria	NG3	2022	1551	29	1.87	1.305	2.672	3.4	1551	2	0.129	0.035	0.469	1.047	1551	1	0.064	0.003	0.364	NA	1551	222	14.313	12.659	16.144	9.9	1551	190	12.25	10.711	13.976	NA	1551	28	1.805	1.252	2.597	NA	1551	4	0.258	0.1	0.661	NA	1551	55	3.546	2.734	4.587	2.249
Nigeria	NG4	2019	1049	63	6.006	4.722	7.61	3.4	1049	1	0.095	0.005	0.538	1.047	1049	7	0.667	0.324	1.371	NA	1049	322	30.696	27.979	33.553	9.9	1049	314	29.933	27.239	32.774	NA	1049	6	0.572	0.262	1.242	NA	1049	2	0.191	0.052	0.692	NA	1049	44	4.194	3.139	5.584	2.249

Nigeria	NG4	2020	798	25	3.133	2.131	4.584	3.4	798	2	0.251	0.069	0.909	1.047	798	2	0.251	0.069	0.909	NA	798	96	12.03	9.953	14.471	9.9	798	94	11.779	9.724	14.201	NA	798	0	0	0	0.479	NA	798	2	0.251	0.069	0.909	NA	798	44	5.514	4.133	7.321	2.249
Nigeria	NG4	2021	815	49	6.012	4.577	7.86	3.4	815	2	0.245	0.067	0.89	1.047	815	1	0.123	0.006	0.692	NA	815	83	10.184	8.291	12.451	9.9	815	67	8.221	6.525	10.308	NA	815	14	1.718	1.026	2.863	NA	815	2	0.245	0.067	0.89	NA	815	32	3.926	2.795	5.49	2.249
Nigeria	NG4	2022	740	55	7.432	5.755	9.55	3.4	740	0	0	0	0.516	1.047	740	6	0.811	0.372	1.758	NA	740	56	7.568	5.873	9.7	9.9	740	48	6.486	4.927	8.495	NA	740	7	0.946	0.459	1.94	NA	740	1	0.135	0.007	0.761	NA	740	38	5.135	3.764	6.97	2.249
Tanzania	TZ1	2019	1585	0	0	0	0.242	2	1585	0	0	0	0.242	0.238	0	0	NA	NA	NA	NA	0	0	NA	NA	NA	8.4	0	0	NA	NA	NA	NA	0	0	NA	NA	NA	NA	0	0	NA	NA	NA	NA	1585	0	0	0	0.242	1.828
Tanzania	TZ1	2020	1798	0	0	0	0.213	2	1798	0	0	0	0.213	0.238	0	0	NA	NA	NA	NA	0	0	NA	NA	NA	8.4	0	0	NA	NA	NA	NA	0	0	NA	NA	NA	NA	0	0	NA	NA	NA	NA	1798	0	0	0	0.213	1.828
Tanzania	TZ1	2021	2083	0	0	0	0.184	2	2083	0	0	0	0.184	0.238	0	0	NA	NA	NA	NA	0	0	NA	NA	NA	8.4	0	0	NA	NA	NA	NA	0	0	NA	NA	NA	NA	0	0	NA	NA	NA	NA	2083	0	0	0	0.184	1.828
Tanzania	TZ1	2022	2716	0	0	0	0.141	2	2716	0	0	0	0.141	0.238	0	0	NA	NA	NA	NA	0	0	NA	NA	NA	8.4	0	0	NA	NA	NA	NA	0	0	NA	NA	NA	NA	0	0	NA	NA	NA	NA	2716	0	0	0	0.141	1.828
Tanzania	TZ2	2019	122	0	0	0	3.053	2	122	0	0	0	3.053	0.238	122	0	0	0	3.053	NA	122	0	0	0	3.053	8.4	122	0	0	0	3.053	NA	122	0	0	0	3.053	NA	122	0	0	0	3.053	NA	122	3	2.459	0.84	6.981	1.828
Tanzania	TZ2	2020	788	2	0.254	0.07	0.921	2	788	0	0	0	0.485	0.238	788	0	0	0	0.485	NA	788	0	0	0	0.485	8.4	788	0	0	0	0.485	NA	788	0	0	0	0.485	NA	788	0	0	0	0.485	NA	788	4	0.508	0.198	1.298	1.828
Tanzania	TZ2	2021	1084	2	0.185	0.051	0.67	2	1084	0	0	0	0.353	0.238	1084	0	0	0	0.353	NA	1084	0	0	0	0.353	8.4	1084	0	0	0	0.353	NA	1084	0	0	0	0.353	NA	1084	0	0	0	0.353	NA	1084	3	0.277	0.094	0.811	1.828
Tanzania	TZ2	2022	958	1	0.104	0.005	0.589	2	958	0	0	0	0.399	0.238	958	0	0	0	0.399	NA	958	0	0	0	0.399	8.4	958	0	0	0	0.399	NA	958	0	0	0	0.399	NA	958	0	0	0	0.399	NA	958	6	0.626	0.287	1.36	1.828
Tanza	TZ3	2019	1225	0	0	0	0.3	2	1139	0	0	0	0.3	0.2	112	0	0	0	3.3	NA	112	0	0	0	3.3	8.4	112	0	0	0	3.3	NA	112	0	0	0	3.3	NA	112	0	0	0	3.3	NA	1225	0	0	0	0.3	1.8

nia							13						36	38					16						16						16						16						16						13	28
Tanzania	TZ3	2020	1652	0	0	0	0.232	2	1652	0	0	0	0.232	0.238	140	0	0	0	2.671	NA	140	0	0	0	2.671	8.4	140	0	0	0	2.671	NA	140	0	0	0	2.671	NA	140	0	0	0	2.671	NA	1652	0	0	0	0.232	1.828
Tanzania	TZ3	2021	1538	0	0	0	0.249	2	1438	0	0	0	0.266	0.238	0	0	NA	NA	NA	NA	0	0	NA	NA	NA	8.4	0	0	NA	NA	NA	NA	0	0	NA	NA	NA	NA	0	0	NA	NA	NA	NA	1538	0	0	0	0.249	1.828
Tanzania	TZ3	2022	1348	0	0	0	0.284	2	1348	0	0	0	0.284	0.238	0	0	NA	NA	NA	NA	0	0	NA	NA	NA	8.4	0	0	NA	NA	NA	NA	0	0	NA	NA	NA	NA	0	0	NA	NA	NA	NA	1348	0	0	0	0.284	1.828
Tanzania	TZ4	2019	1076	0	0	0	0.356	2	1076	0	0	0	0.356	0.238	0	0	NA	NA	NA	NA	0	0	NA	NA	NA	8.4	0	0	NA	NA	NA	NA	0	0	NA	NA	NA	NA	0	0	NA	NA	NA	NA	1076	3	0.279	0.095	0.817	1.828
Tanzania	TZ4	2020	1582	0	0	0	0.242	2	1582	0	0	0	0.242	0.238	0	0	NA	NA	NA	NA	0	0	NA	NA	NA	8.4	0	0	NA	NA	NA	NA	0	0	NA	NA	NA	NA	0	0	NA	NA	NA	NA	1582	9	0.569	0.3	1.078	1.828
Tanzania	TZ4	2021	2562	0	0	0	0.15	2	2562	1	0.039	0.002	0.221	0.238	0	0	NA	NA	NA	NA	0	0	NA	NA	NA	8.4	0	0	NA	NA	NA	NA	0	0	NA	NA	NA	NA	0	0	NA	NA	NA	NA	2328	1	0.043	0.002	0.243	1.828
Tanzania	TZ4	2022	2840	0	0	0	0.135	2	2840	1	0.035	0.002	0.129	0.238	0	0	NA	NA	NA	NA	0	0	NA	NA	NA	8.4	0	0	NA	NA	NA	NA	0	0	NA	NA	NA	NA	0	0	NA	NA	NA	NA	2840	3	0.106	0.036	0.31	1.828
Tanzania	TZ5	2019	2153	1	0.046	0.002	0.263	2	2153	1	0.046	0.002	0.263	0.238	2153	0	0	0	0.178	NA	0	0	NA	NA	NA	8.4	0	0	NA	NA	NA	NA	2153	0	0	0	0.178	NA	2153	0	0	0	0.178	NA	2153	9	0.418	0.22	0.793	1.828
Tanzania	TZ5	2020	2142	0	0	0	0.179	2	2142	0	0	0	0.179	0.238	2142	1	0.047	0.002	0.264	NA	0	0	NA	NA	NA	8.4	0	0	NA	NA	NA	NA	2142	0	0	0	0.179	NA	2142	0	0	0	0.179	NA	2142	6	0.28	0.128	0.61	1.828
Tanzania	TZ5	2021	2493	3	0.12	0.041	0.353	2	2493	0	0	0	0.154	0.238	2493	2	0.08	0.022	0.292	NA	0	0	NA	NA	NA	8.4	0	0	NA	NA	NA	NA	2493	0	0	0	0.154	NA	2493	0	0	0	0.154	NA	2493	7	0.281	0.136	0.578	1.828
Tanzania	TZ5	2022	2675	3	0.112	0.038	0.329	2	2675	3	0.112	0.038	0.329	0.238	2675	0	0	0	0.143	NA	0	0	NA	NA	NA	8.4	0	0	NA	NA	NA	NA	2675	0	0	0	0.143	NA	2675	0	0	0	0.143	NA	2675	4	0.15	0.058	0.384	1.828

Uganda	UG1	2019	210	0	0	0	1.796	1.8	210	0	0	0	1.796	0.284	210	0	0	0	1.796	NA	210	0	0	0	1.796	10	210	0	0	0	1.796	NA	210	0	0	0	1.796	NA	210	0	0	0	1.796	NA	210	4	1.905	0.743	4.794	1.51
Uganda	UG1	2020	246	0	0	0	1.538	1.8	246	0	0	0	1.538	0.284	246	0	0	0	1.538	NA	246	1	0.407	0.021	2.266	10	246	1	0.407	0.021	2.266	NA	246	0	0	0	1.538	NA	246	0	0	0	1.538	NA	246	1	0.407	0.021	2.266	1.51
Uganda	UG1	2021	326	0	0	0	1.165	1.8	326	0	0	0	1.165	0.284	326	0	0	0	1.165	NA	326	14	4.294	2.575	7.078	10	326	14	4.294	2.575	7.078	NA	326	0	0	0	1.165	NA	326	0	0	0	1.165	NA	326	0	0	0	1.165	1.51
Uganda	UG1	2022	238	0	0	0	1.588	1.8	238	0	0	0	1.588	0.284	238	0	0	0	1.588	NA	238	1	0.42	0.022	2.341	10	238	0	0	0	1.588	NA	238	1	0.42	0.022	2.341	NA	238	0	0	0	1.588	NA	238	1	0.42	0.022	2.341	1.51
Uganda	UG2	2019	1443	5	0.347	0.148	0.809	1.8	1443	0	0	0	0.266	0.284	1443	0	0	0	0.266	NA	1443	149	10.326	8.86	12.003	10	1443	136	9.425	8.023	11.042	NA	1443	9	0.624	0.328	1.181	NA	1443	4	0.277	0.108	0.711	NA	1443	15	1.04	0.631	1.708	1.51
Uganda	UG2	2020	1328	5	0.377	0.161	0.878	1.8	1328	1	0.075	0.004	0.425	0.284	1328	0	0	0	0.288	NA	1328	59	4.443	3.46	5.689	10	1328	49	3.69	2.802	4.845	NA	1328	7	0.527	0.256	1.084	NA	1328	3	0.226	0.067	0.662	NA	1328	6	0.452	0.207	0.982	1.51
Uganda	UG2	2021	409	2	0.489	0.134	1.765	1.8	409	1	0.244	0.013	1.372	0.284	409	0	0	0	0.93	NA	409	13	3.178	1.867	5.362	10	409	13	3.178	1.867	5.362	NA	409	0	0	0	0.93	NA	409	0	0	0	0.93	NA	409	9	2.2	1.162	4.129	1.51
Uganda	UG2	2022	3216	14	0.435	0.259	0.729	1.8	3216	1	0.031	0.002	0.176	0.284	3216	3	0.093	0.032	0.274	NA	3216	249	7.743	6.868	8.717	10	3216	228	7.09	6.253	8.029	NA	3216	15	0.466	0.283	0.768	NA	3216	6	0.187	0.086	0.406	NA	3216	30	0.933	0.654	1.329	1.51
Uganda	UG3	2019	6932	23	0.332	0.221	0.497	1.8	6932	4	0.058	0.022	0.148	0.284	6932	5	0.072	0.031	0.169	NA	6932	302	4.357	3.901	4.863	10	6932	286	4.126	3.682	4.62	NA	6932	16	0.231	0.142	0.375	NA	6932	0	0	0	0.055	NA	6932	38	0.548	0.4	0.751	1.51
Uganda	UG3	2020	6281	20	0.318	0.206	0.491	1.8	6281	2	0.032	0.009	0.116	0.284	6281	8	0.127	0.065	0.251	NA	6281	303	4.824	4.321	5.382	10	6281	285	4.537	4.05	5.081	NA	6281	18	0.287	0.181	0.453	NA	6281	0	0	0	0.061	NA	6281	37	0.589	0.428	0.811	1.51
Uganda	UG3	2021	5501	21	0.382	0.25	0.583	1.8	5501	3	0.055	0.019	0.16	0.284	5501	5	0.091	0.039	0.213	NA	5501	328	5.963	5.367	6.62	10	5501	318	5.781	5.194	6.429	NA	5501	10	0.182	0.099	0.334	NA	5501	0	0	0	0.07	NA	5501	34	0.618	0.443	0.862	1.51
Uga	UG3	20	64	19	0.2	0.1	0.4	1.8	64	4	0.0	0.0	0.	0.2	64	5	0.0	0.0	0.1	NA	64	344	5.3	4.8	5.9	10	64	330	5.1	4.6	5.7	NA	64	12	0.1	0.1	0.3	NA	64	2	0.0	0.0	0.1	NA	64	30	0.4	0.3	0.6	1.

nda		22	24		96	89	62		24		62	24	16	84	24		78	33	82		24		55	31	33		24		37	23	04		24		87	07	26		24		31	09	13		24		67	27	66	51
Uganda	UG4	2019	4820	3	0.062	0.021	0.183	1.8	4820	0	0	0	0.08	0.284	4820	2	0.041	0.011	0.151	NA	4820	354	7.344	6.641	8.115	10	4820	336	6.971	6.286	7.725	NA	4820	11	0.228	0.127	0.408	NA	4820	7	0.145	0.07	0.299	NA	4820	14	0.29	0.173	0.487	1.51
Uganda	UG4	2020	7499	0	0	0	0.051	1.8	7499	0	0	0	0.051	0.284	7499	2	0.027	0.007	0.097	NA	7499	405	5.401	4.912	5.936	10	7499	372	4.961	4.492	5.476	NA	7499	21	0.28	0.183	0.428	NA	7499	12	0.16	0.092	0.28	NA	7499	24	0.32	0.215	0.476	1.51
Uganda	UG4	2021	7051	0	0	0	0.054	1.8	7051	0	0	0	0.054	0.284	7051	2	0.028	0.008	0.103	NA	7051	326	4.623	4.158	5.139	10	7051	290	4.113	3.674	4.602	NA	7051	24	0.34	0.229	0.506	NA	7051	12	0.17	0.097	0.297	NA	7051	24	0.34	0.229	0.506	1.51
Uganda	UG4	2022	7217	0	0	0	0.053	1.8	7217	0	0	0	0.053	0.284	7217	6	0.083	0.038	0.181	NA	7217	411	5.695	5.183	6.253	10	7217	381	5.279	4.787	5.819	NA	7217	17	0.236	0.147	0.377	NA	7217	13	0.18	0.105	0.308	NA	7217	22	0.305	0.201	0.461	1.51
